# Estimating the risk of SARS-CoV-2 infection in New Zealand border arrivals

**DOI:** 10.1186/s44263-024-00057-2

**Published:** 2024-05-03

**Authors:** Richard Arnold, Rachelle N. Binny, Thomas Lumley, Audrey Lustig, Matthew Parry, Michael J. Plank

**Affiliations:** 1 School of Mathematics and Statistics, Victoria University of Wellington, Wellington, New Zealand; 2 Manaaki Whenua - Landcare Research, Lincoln, New Zealand; 3 Department of Statistics, University of Auckland, Auckland, New Zealand; 4 Department of Mathematics and Statistics, University of Otago, Dunedin, New Zealand; 5 School of Mathematics and Statistics, University of Canterbury, Christchurch, New Zealand

**Keywords:** Covid-19, Border control, Mixed Models, Risk assessment

## Abstract

**Background:**

Travel restrictions and border controls were used extensively during the COVID-19 pandemic. However, the processes for making robust evidence-based risk assessments of source countries to inform border control policies was in many cases very limited.

**Methods:**

Between April 2020 and February 2022, all international arrivals to New Zealand were required to spend 14 days in government-managed quarantine facilities and were tested at least twice. The infection rates among arrivals in the years 2020, 2021 and 2022 were respectively 6.3, 9.4 and 90.0 cases per thousand arrivals (487, 1064 and 1496 cases). Test results for all arrivals were linked with travel history, providing a large and comprehensive dataset on the number of SARS-CoV-2-positive and negative travellers from different countries over time. We developed a statistical model to predict the country-level infection risk based on infection rates among recent arrivals and reported cases in the country of origin. The model incorporates a country-level random effect to allow for the differences between the infection risk of the population of each country and that of travellers to New Zealand. A time dependent auto-regressive component of the model allows for short term correlation in infection rates.

**Results:**

A model selection and checking exercise found that the model was robust and reliable for forecasting arrival risk for 2 weeks ahead. We used the model to forecast the number of infected arrivals in future weeks and categorised countries according to their risk level. The model was implemented in R and was used by the New Zealand Ministry of Health to help inform border control policy during 2021.

**Conclusions:**

A robust and practical forecasting tool was developed for forecasting infection risk among arriving passengers during a period of controlled borders during the COVID-19 pandemic. The model uses historical infection rates among arrivals and current infection rates in the source country to make separate risk predictions for arrivals from each country.

**Supplementary Information:**

The online version contains supplementary material available at 10.1186/s44263-024-00057-2.

## Background

Travel restrictions and border controls were used by many countries during the COVID-19 pandemic in an attempt to reduce the number of SARS-CoV-2 infections imported from other jurisdictions. Examples include pre- and post-travel testing, vaccine passports, quarantine of inbound travellers, use of managed isolation facilities and reactive travel bans on specific countries [1–3]. Travel restrictions have had various aims, including limiting community prevalence of SARS-CoV-2, reducing the need for non-pharmaceutical interventions domestically, preventing or reducing the importation of a variant of concern, or maintaining a state of SARS-CoV-2 elimination.

The effectiveness of travel restrictions varied depending on factors such as the prevalence of SARS-CoV-2 (or a particular variant) in the origin and destination localities, level of compliance and ability to enforce restrictions, risk of transmission while in transit or within managed isolation facilities, and volume of travellers [4–6]. Evaluating the effectiveness of specific interventions is crucial for three key reasons: (1) to enable governments to make evidence-based decisions about which interventions to use in specific circumstances; (2) to make sure measures are equitable, legally and politically justifiable with respect to a stated public health aim; and (3) so that measures can be targeted to high-risk cohorts of travellers rather than applied in an arbitrary or blanket manner [7]. These considerations also apply to future pandemic threats which may prompt governments to impose travel restrictions.

Modelling studies have been used extensively to estimate the effectiveness of different sets of border controls [8–12]. However, the global evidence base for quantifying the risk posed by arriving travellers in different situations is lacking [13]. This is partly due to a lack of systematically collected data on cohorts of travellers and partly due to variability of testing policies and of the quality of official data across jurisdictions and through time.

Between April 2020 and February 2022, New Zealand required all international arrivals, with limited exceptions, to stay in government-managed isolation and quarantine (MIQ) facilities for 14 days [2]. Due to the isolated location of New Zealand in the South Pacific the vast majority of international arrivals are by air and, as a result of fewer flight routes and reduced demand for travel during the pandemic, travellers arrived almost exclusively at the country’s two largest international airports (Auckland and Christchurch). This enabled New Zealand to implement border control measures that were uniform and comprehensive.

The use of MIQ was a key part of a strategy to eliminate community transmission of SARS-CoV-2, or suppress it to very low levels until high vaccination rates were achieved in late 2021 [14, 15]. During this period, all international arrivals were tested at least twice and, from January 2021, three times during their stay in MIQ (on days 0, 3 and 12 after arrival). From January 2021, travellers were additionally required to provide a negative PCR test taken up to 72 h prior to departure. These strict border controls created a unique dataset of more than 200,000 inbound travellers which can be used to evaluate SARS-CoV-2 infection risk in arrivals from different countries and at different stages in the pandemic.

Throughout the pandemic the New Zealand Government sought a robust means of assessing the risk of importing cases of COVID-19 via international travel. The level of risk depends on several factors including the COVID-19 incidence rate, vaccine coverage and public health measures in the country of origin and any transit countries, and the risk of in-flight transmission along the route taken from country of origin to New Zealand.

These risk assessments were important for two main reasons. Firstly, the risk of onward transmission of SARS-CoV-2 into the community, for example via infection of MIQ workers or arrivals still being infectious after release from MIQ, will generally increase with the number of infected arrivals [2]. Secondly, positive cases detected in MIQ were moved to a dedicated isolation facility with limited capacity. Having a large number of infected arrivals in a short period of time could cause this capacity to be exceeded. In some instances, additional travel restrictions were imposed on travellers from countries designated as very high risk. Conversely, countries designated as low risk were at times subject to more relaxed measures, such as self-isolation at home or quarantine-free travel.

Kucharski et al. [16] developed a mathematical model to use data from testing of inbound travellers to infer prevalence in the country of origin in real time. Their method adjusts for the effect of pre-departure testing requirements, which reduce the apparent prevalence among inbound travellers by preventing some infected individuals from travelling. In principle, this approach could be used to reconstruct global transmission dynamics from traveller screening data. Some countries had prevalence estimates from community sampling studies, notably the UK’s Office for National Statistics COVID-19 infection survey [17] or REACT study [18]. However, these estimates were not available for most jurisdictions and country-specific estimates of infection prevalence would fill a key gap in global pandemic surveillance.

Quilty et al. [19] estimated the transmission risk posed by international travellers based on number of passengers and estimated incidence of SARS-CoV-2 in different countries. Their travel volume estimates were based on the OpenSky database [20] and incidence estimates were based on reported deaths and an assumed infection fatality ratio [9, 21]. There are limitations to both these estimates and it would be preferable to use data on actual travel numbers and prevalence of infection among travellers where available.

In this paper, we develop a statistical model that can be deployed in real time to estimate the probability that a future arrival from a given country is infected with SARS-CoV-2. The model is trained on data on confirmed cases of COVID-19 detected in recently arrived travellers to New Zealand, the numbers of arrivals from different countries over a 30-week period, and publicly accessible data obtained from the Johns Hopkins COVID-19 Data Repository via Our World In Data [22].

Through its calibration on actual arriving cases, the model accounts for possible variations in case reporting between countries. Where a country has a high level of unreported infection, the rate of infection among arrivals may be higher than official data from that country would suggest. Conversely, in countries where the incidence of infection is low and mostly confined to inbound travel-related cases, then the rate of infection in arrivals from that country may be lower than expected. The model uses recent trends to account for these effects and adjust estimates of future risk via a country-specific, time-varying component.

We do not attempt to reconstruct prevalence estimates for country of origin as done by Kucharski et al. [16]. Rather, our aim is to provide a robust framework for estimating changing levels of infection risk in inbound travellers within a short time horizon of only a few weeks. This paper makes two main contributions: one is a quantitative assessment of how infection risk in travellers varied in relation to reported cases of COVID-19 in the country of origin and at different stages in the pandemic. The second main contribution is to provide a robust statistical framework that can be used by policymakers in real time to assess risk. This framework was used by the New Zealand government to inform decisions around border controls and border reopening strategy throughout 2021 and early 2022.

A previous non peer-reviewed technical report describing this work has already been published which describes the data and analysis in this manuscript [23].

## Methods

### Data

Daily counts of arrivals into New Zealand by country of origin were obtained from StatsNZ based on passenger arrival card data. Out of *n* = 207,518 arrivals between 8 June 2020 and 20 February 2022, there were 4060 (2.0%) whose arrival card country of origin could not be matched to a standard country name (see Additional file 1: Supplementary data description), or where the country did not have corresponding data in the Our World in Data dataset. These arrivals were assigned to ‘Unknown origin’.

Daily counts of detected cases of COVID-19 in recent international arrivals by country of origin were provided by the New Zealand Ministry of Health (ESR, EpiSurv dataset, [24]). Cases were included if their *Status* field was ‘Confirmed’ or ‘Probable’ and the *Overseas* field was ‘Yes’, and were recorded by date of arrival to New Zealand rather than date of positive test to enable comparison with arrival counts. This give a total *n* = 3047 cases arriving at the border between 8 June 2020 and 20 February 2022. Country of origin was derived from the passenger Arrival Card, which is completed by all arriving travellers. Where a reliable country of origin could not be identified from the Arrival Card the last known port of origin along the passenger’s route was used. The country of origin of positive cases reported in the EpiSurv database is only used if no other country of origin is available. Although the EpiSurv data source is likely to be the most reliable for country of origin, it is only available for cases, and its use would introduce an undesirable numerator (cases)/denominator (all arrivals) mismatch. There were 2 cases (0.1%) with unknown origin.

International data on new daily confirmed cases, confirmed deaths, estimated effective reproduction number $R_\text {eff}$, partial and full vaccination coverage, number of tests, and test positivity rate were obtained from the Our World in Data COVID-19 dataset [22]. Occasional negative counts of cases, deaths or tests in this dataset were set to zero. Estimates of $R_\text {eff}$ were also obtained from Epiforecasts [25].

Data were aggregated to weekly totals (Monday–Sunday) before modelling. This reduced the proportion of observations of very small counts, as well as eliminating day-of-the-week effects in reporting. If the final week of data had 5 or 6 days, we scaled the number of cases and arrivals so that the counts are equivalent to a weekly total. If the final week had fewer than 5 days of data, all data for that week were excluded.

### Statistical model

We modelled the number of COVID-19 cases $Y_{ct}$ detected out of $N_{ct}$ arrivals from country *c* in time period *t* using a Binomial count model

1 $$\begin{aligned} Y_{ct} \sim \text {Binomial}(N_{ct}, \mu _{ct}), \end{aligned}$$

where $\mu _{ct}$ is the probability of an arrival from country *c* in time period *t* being infected with SARS-CoV-2. For $\mu _{ct}$, we used a mixed effects model with the lagged number of per capita weekly reported cases $I_{ct}$ (which we will refer to as the case rate) in origin country *c* as the only predictor. This predictor was chosen following a model selection procedure using a set of candidate predictor variables (confirmed COVID-19 deaths, number of tests, test positivity rate, proportion of the population at least partially vaccinated, proportion fully vaccinated, effective reproduction number) — see Additional file 1: Supplementary methods for details. We found that most of the candidate variables were either too incomplete or too weakly informative to be useful as predictors. In particular, testing rates and vaccination rates were too strongly varying over time, due to changing policies in the country of origin, that they could not be used with confidence in a predictive model.

The model for $\mu _{ct}$ was

2 $$\begin{aligned} \text {logit} \mu _{ct} = \alpha + \sum _{k=k_1}^{k_2} \beta _k \text {logit} \left( \delta + I_{c,t-k}\right) + u_{c} + v_{ct}, \end{aligned}$$

where $\alpha$ is an intercept, $\beta _{k_1},\ldots ,\beta _{k_2}$ are regression coefficients associated with the lagged logit-transformed case rates $I_{t-k_1},\ldots ,I_{t-k_2}$; $u_c \overset{\text {iid}}{\sim } N(0,\sigma _u^2)$ is a country-level random effect; $v_{ct} = \rho v_{c,t-1} + \varepsilon _{ct}$ is an autoregressive AR(1) error structure with correlation parameter $\rho$ and error term $\varepsilon _{ct} \overset{\text {iid}}{\sim } N(0,\sigma _e^2)$; and $\delta =10^{-7}$ is a small offset to case rate observations which allows for situations where the number of reported cases is 0. Our model selection process selected lags $k_1=0$ to $k_2=2$ as optimal.

The random effects structure assigns a country-level random effect $u_c$ to country *c* to account for arrivals from that country differing in risk from the risk level suggested by the reported case rate $I_{ct}$. The autoregressive error structure $v_{ct}$ allows this country-level effect to change over time with temporal correlation.

We fitted the model using the R package glmmTMB [26], which implements the TMB package [27] for use with generalised linear mixed effects models. For each forecast, we restricted the model training data to a fixed time window, the most recent 30 weeks, to allow for long-term changes in the pandemic.

Given data on border cases, arrivals, and case rate in country of origin $(Y_{ct}, N_{ct}, I_{ct})$ at *n* weekly time points $(t_1,\ldots ,t_n)$ and countries $c=1,\ldots ,C$, the TMB estimation function returns parameter estimates $\widehat{\varvec{\beta }}$, $\widehat{\rho }$, $\widehat{\sigma }^2_u$ and $\widehat{\sigma }^2_e$ and their covariances; estimated country-level random effects $\widehat{u}_c$ and time-dependent random effects $\widehat{v}_{ct}$; and fitted values and variance of the logit-transformed probability of infection $\widehat{\eta }_{ct} = \textrm{logit} \ \widehat{\mu }_{ct}$ and the number of positive cases $\widehat{Y}_{ct}=N_{ct}\widehat{\mu }_{ct}$. Confidence intervals and in-sample prediction intervals were constructed using standard methods — see Additional file 1: Supplementary methods for details.

### Forecasts

To forecast the model a further *k* time steps beyond the last observation $t_n$, we required first a method for forecasting the number of arrivals $N_{ct}$ and the reported case rate in the country of origin $I_{ct}$. We forecast arrivals $N_{ct}$ using data on MIQ bookings. Where these were not available, we set $N_{ct}$ for all $t>t_n$ to be the mean number of arrivals $\bar{N}_c$ for country *c* in a fixed time window prior to the last observation at $t_n$. We forecast reported cases in the country of origin using a weighted linear fit to $\text {logit}(\delta +I_{ct})$ using the last $m=3$ observations with weights $1/m, 2/m, \ldots , m/m$. We do not include any quantitative estimate of the effect of uncertainties in the forecasts of $N_{ct}$ and $I_{ct}$, but we do discuss the impact of these uncertainties in the Results section.

The only time-varying component of the prediction model in Eqs. (1) and (2) is the autoregressive random effect $v_{ct}$. We projected these *k* steps forward via $\widehat{v}_{ct_{n+k}} = \widehat{\rho }^k v_{ct_n} + \zeta _{ct_{n+k}}$, where $\zeta _{ct_{n+k}} \overset{\text {iid}}{\sim } N\left( 0,\widehat{\sigma }_e^2 (1-\widehat{\rho }^{2k})/(1-\widehat{\rho }^2)\right)$. Forecasts use the expected value $\widehat{v}_{ct_{n+k}} = \widehat{\rho }^k v_{ct_n}$.

Note that these confidence and prediction intervals neglect any uncertainty introduced in the forecasting of the numbers of arrivals and of the reported case rate in the country of origin. The number of arrivals may be estimated from MIQ bookings or, for instances where passengers are not required to use MIQ, could be estimated from flight schedules.

We also calculated one step ahead forecasts as a useful measure of model goodness of fit, see Additional file 1: Supplementary methods.

### Low information countries

The model cannot provide good estimates for countries where there is low information, either due to low numbers of arrivals, low numbers of cases, or both. We fitted the full model only for countries with 50 or more arrivals and 5 or more cases in the most recent 30 weeks.

For the remaining countries where there has been at least one recent arrival ($\sum _t N_{ct}>0$) and at least one case ($\sum _t Y_{ct}>0$), we fit a simpler model without the autoregressive AR(1) component in (2). For all other countries, we simply estimate the logit-transformed infection probability $\mu _{ct}$ using the regression coefficients $\widehat{\beta }$ from the main model fit without any country-level or time-varying effects — see Additional file 1: Supplementary methods for details.

### Risk classification

In each time period *t*, we classified each country via a multi-level risk categorisation using the fitted and forecasted estimates of cases $\widehat{Y}_{ct}$ and infection probability $\widehat{\mu }_{ct}$. A risk classification for country *c* at time *t* can be made according to one of (a) the expected number of cases arriving at the border $\widehat{Y}_{ct}$, (b) the upper bound of the confidence interval for $\widehat{Y}_{ct}$, or (c) the upper bound of the prediction interval for $Y_{ct}$. Risk thresholds based on confidence intervals allow for the uncertainty in the estimate of $\widehat{Y}_{ct}$. Risk thresholds based on prediction intervals allow for both the uncertainty in the estimate of $\widehat{Y}_{ct}$ and the uncertainty in the observed value of the random variable $Y_{ct}$.

Risk classification based on the expected number of cases $\widehat{Y}_{ct}$ requires some knowledge of the expected number of arrivals $N_{ct}$. An alternative risk classification can be based only on the infection probability $\widehat{\mu }_{ct}$ instead, again using either the point estimate for $\widehat{\mu }_{ct}$, the confidence interval, or the prediction interval. However, calculation of the prediction interval for the observed proportion of arrivals that are infected, $Y_{ct}/N_{ct}$, still requires an estimate of the numbers of future arrivals $N_{ct}$.

If confidence or prediction intervals are used, it is suitable to use confidence intervals with confidence levels of the order of 50% (rather than, say 95%), i.e. where the upper bound of the interval is the upper quartile. This avoids overly conservative risk classifications. For illustrative purposes in this paper, we have used a risk classification scheme with four risk classes using the upper bound of the 50% confidence interval for the infection probability, with cut-points of 3, 8 and 20 cases per thousand arrivals.

### Assessing model fit

There are a number of ways to assess how well the model fits the data, and how well reliable the forecasts are. In the model selection process we used the AIC criterion and the mean absolute deviation (MAD) of one step ahead forecasts of cases to assess the fit of a set of candidate models in two independent data sets (see Additional file 1: Supplementary methods).

Out of sample assessments of goodness of fit can be made by comparing the forecasts of cases and rates with the actual values. This comparison can only be made for instances where there were actual arrivals in the forecast period. Forecasts were made for 224 countries; however, there were arrivals only from 88 countries in the first forecast week, and 89 in the second. In countries with small numbers of arrivals the case numbers are highly volatile, and so we compare using *Z*-scores the estimated cases and rates the forecast for each country in only the first two forecast weeks. The *Z*-scores normalise the difference between the observed case counts $Y_{ct}$ or observed case rate $Y_{ct}/N_{ct}$ and their forecast values by the estimated prediction error:

3 $$\begin{aligned} Z_{\text {cases}}= & {} \frac{Y_{ct}-\widehat{Y}_{ct}}{\text {PE}[\widehat{Y}_{ct}]}\nonumber \\ Z_{\text {rates}}= & {} \frac{\frac{Y_{ct}}{N_{ct}}-\widehat{\mu }_{ct}}{\text {PE}[\widehat{Y}_{ct}]/\widehat{N}_{ct}}\nonumber \\ \text {PE}[\widehat{Y}_{ct}]\simeq & {} \widehat{N}_{ct}\widehat{\mu }_{ct}(1-\widehat{\mu }_{ct}) \sqrt{ \frac{1}{\widehat{N}_{ct}\widehat{\mu }_{ct}(1-\widehat{\mu }_{ct})}+ \text {SE}[\widehat{\eta }_{ct}]^2} \end{aligned}$$

The prediction errors combine the binomial variation $\text {Var}[Y_{ct}|N_{ct},\widehat{\mu }_{ct}]$ with $\text {SE}[\widehat{\eta }_{ct}]$, which is the standard error of the logit transformed rate $\textrm{logit} \widehat{\mu }_{ct}$ derived from the model fit (see Additional file 1: Supplementary methods for further details). The key difference between the case and rate *Z*-scores is that the case score is calculated without reference to the actual number of arrivals $N_{ct}$, whereas the rate score includes this information. We expect these *Z-*scores to follow a standard Normal distribution (mean zero, standard deviation 1) if the data are fitted well by the model. Given that rates are restricted to be non-negative the distribution of *Z*-scores cannot extend to large negative values. However large positive values, which indicate that the actual border rate observed was much higher than the predicted rate, are possible.

### Software implementation and data

The model was implemented in the R software language using the glmmTMB package for model fitting. Although the data presented in this paper are confidential, due to small numbers of arrivals and cases from some countries, the code is available at https://gitlab.com/arnoldri/nzarrivalrisk [28].

## Results

**Fig. 1:**
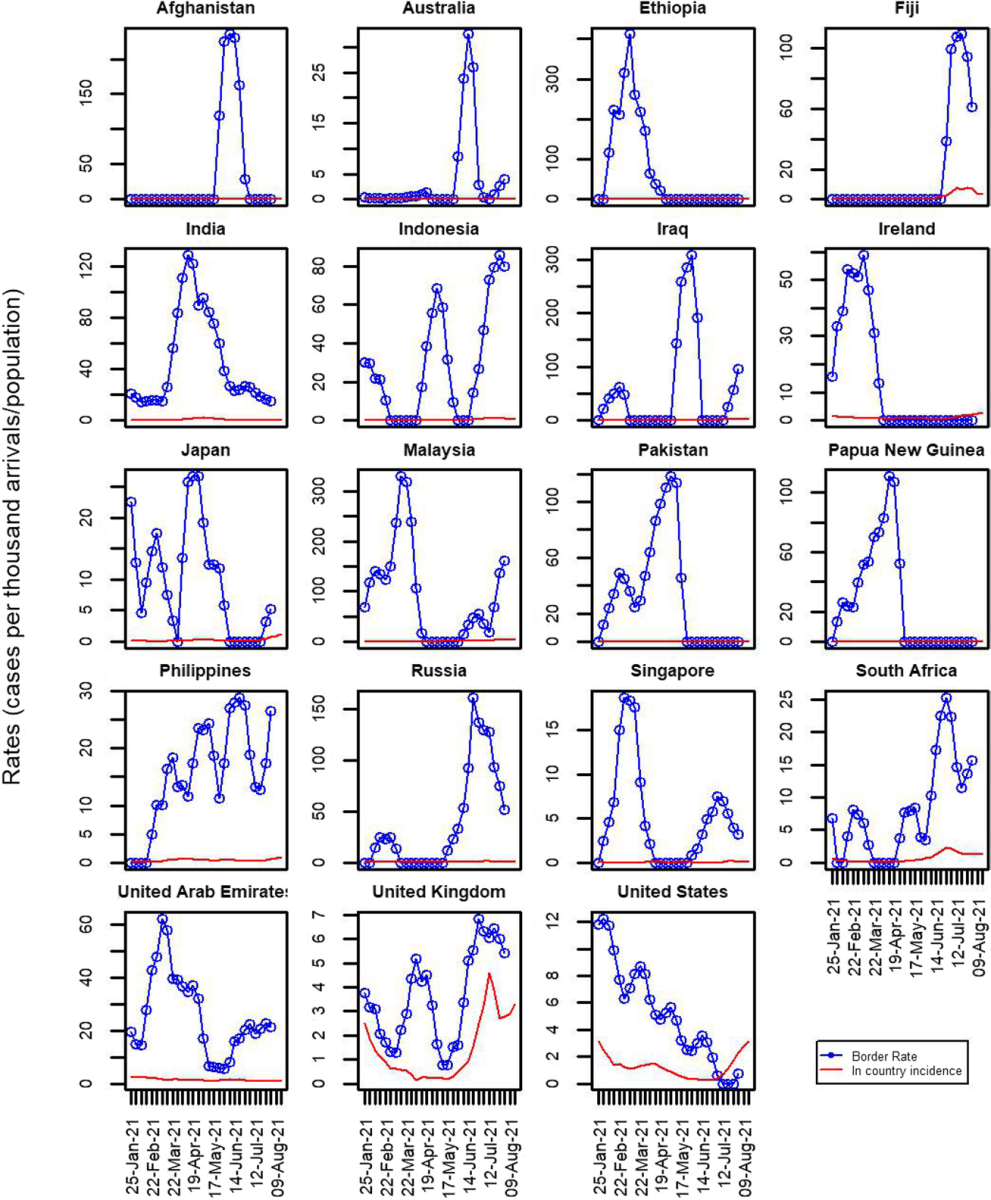
Data on weekly reported cases per thousand in the country of origin (solid red curves) and observed rates of infection among arrivals to New Zealand (blue curves with open circles) in the 19 fully modelled countries. To make these plots clearer, infection rates among arrivals were smoothed using a 5-point smoother for arriving cases (weights $\frac{1}{8}, \frac{1}{4}, \frac{1}{4},\frac{1}{4},\frac{1}{8}$). For an equivalent set of graphs without smoothing, see Additional file 1: Fig. S2

In this section, we show the results for the model fitted to data in the 30-week time interval 25 January 2021 to 22 August 2021. Out of a total of 224 countries, 19 countries had sufficient numbers of arrivals and cases to be included in the full model (see Fig. 1), 44 other countries had at least one 1 case, and 161 had zero cases.

### Fitted model

The parameter estimates and their standard errors are shown in Table 1. The parameter estimates $\widehat{\beta }_0, \widehat{\beta }_1, \widehat{\beta }_2$ quantify the dependence of the infection risk on case rate in the source country at lag 0, 1 and 2 weeks, respectively. For ease of interpretation, we have also transformed the fitted model parameters into alternative representations: $\widehat{\beta }_{\text {ave}}$, the effect of the mean logit-transformed case rate over the last 3 weeks, and parameters $\widehat{\beta }_{1-0}$ and $\widehat{\beta }_{2-1}$ which are the coefficients of the changes in the logit-transformed case rate from lag 1 to lag 0, and lag 2 to lag 1, respectively.

**Table 1:** Parameter estimates for the model fitted to data from 25 January to 22 August 2021

Fitted model parameters^a^			Derived parameters		
Parameter	Estimate	Std. error	Parameter	Estimate	Std. error
$\alpha$	− 1.1648	0.9706	$\alpha$	− 1.1648	0.9706
$\beta _0$	0.2373	0.3080	$\beta _{\text {ave}}$	0.4441	0.1108
$\beta _1$	0.0858	0.4038	$\beta _{1-0}$	0.0892	0.3013
$\beta _2$	0.1210	0.2948	$\beta _{2-1}$	0.0270	0.2905
$\log (\sigma _u)$	− 0.2927	0.3591	$\sigma _u$	0.7462	0.2680
$\log (\sigma _e)$	0.3314	0.1275	$\sigma _e$	1.3929	0.1776
$\theta =\rho /\sqrt{(1-\rho ^2)}$	0.7845	0.2266	$\rho$	0.6172	0.1104

^a^The estimates on the left are returned by the fitting routine, and those on the right are useful transformations of those estimates. Standard errors are calculated using the delta method

We now consider the various aspects of the fitted model, and show results for the 19 fully modelled countries.

**Fig. 2:**
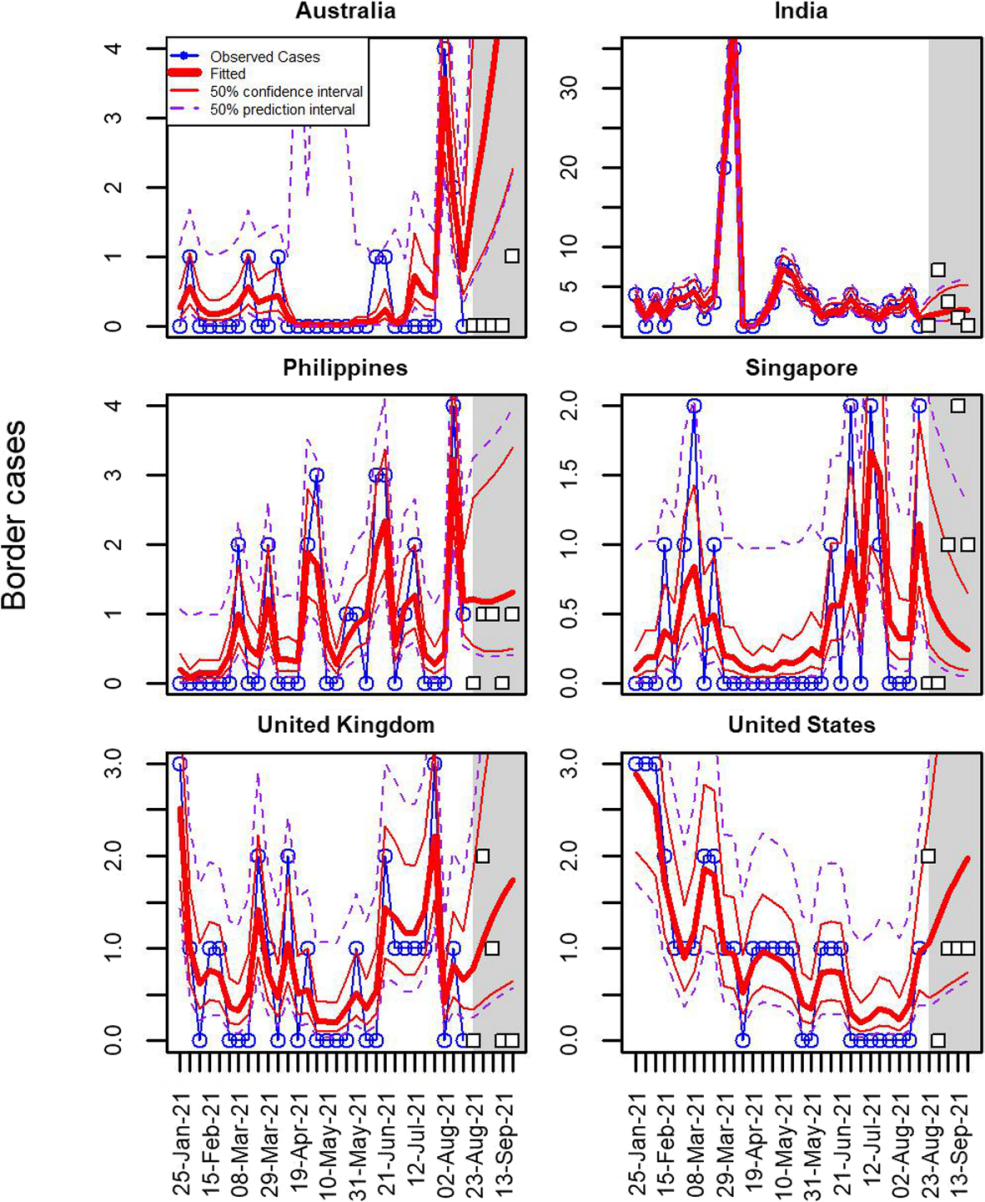
Number of arriving cases from the 6 fully modelled countries with the largest numbers of arrivals (blue). The thick red lines show the fitted model, the thin red lines show the 50% confidence interval around this fit, and the purple dashed lines show the 50% prediction interval. Model fitted to data from 25 January to 22 August 2021. Shaded grey region shows a 12-week forecast period alongside actual data for the first 5 weeks of the forecast period (white squares)

Figure 2 shows the number of infected arrivals at the New Zealand border alongside the fitted model for the six fully modelled countries with the highest number of arrivals. (An equivalent graph for all 19 fully modelled countries is shown in Additional file 1: Fig. S3. In each graph the fitted model is shown as the bold red line with thin red lines bounding the 50% confidence interval and dashed purple lines bounding the 50% prediction interval. The model matches the data well throughout the range of the observations (up to 22 August 2021).

The grey shaded area in Fig. 2 (and in subsequent graphs) shows a 5-week forecast period immediately after the end of the observed data. We only expect the model to provide reliable forecasts for at most a 3-week period, but show 5 weeks to illustrate how the model functions over a slightly longer period. The confidence and prediction intervals expand widely beyond the end of the observed data. Actual observations, not used in the modelling, are shown as open squares in the shaded forecast period.

Additional file 1: Fig. S4 shows an equivalent set of graphs for the infection rate (i.e. number of infections per 1000 arrivals).

**Fig. 3:**
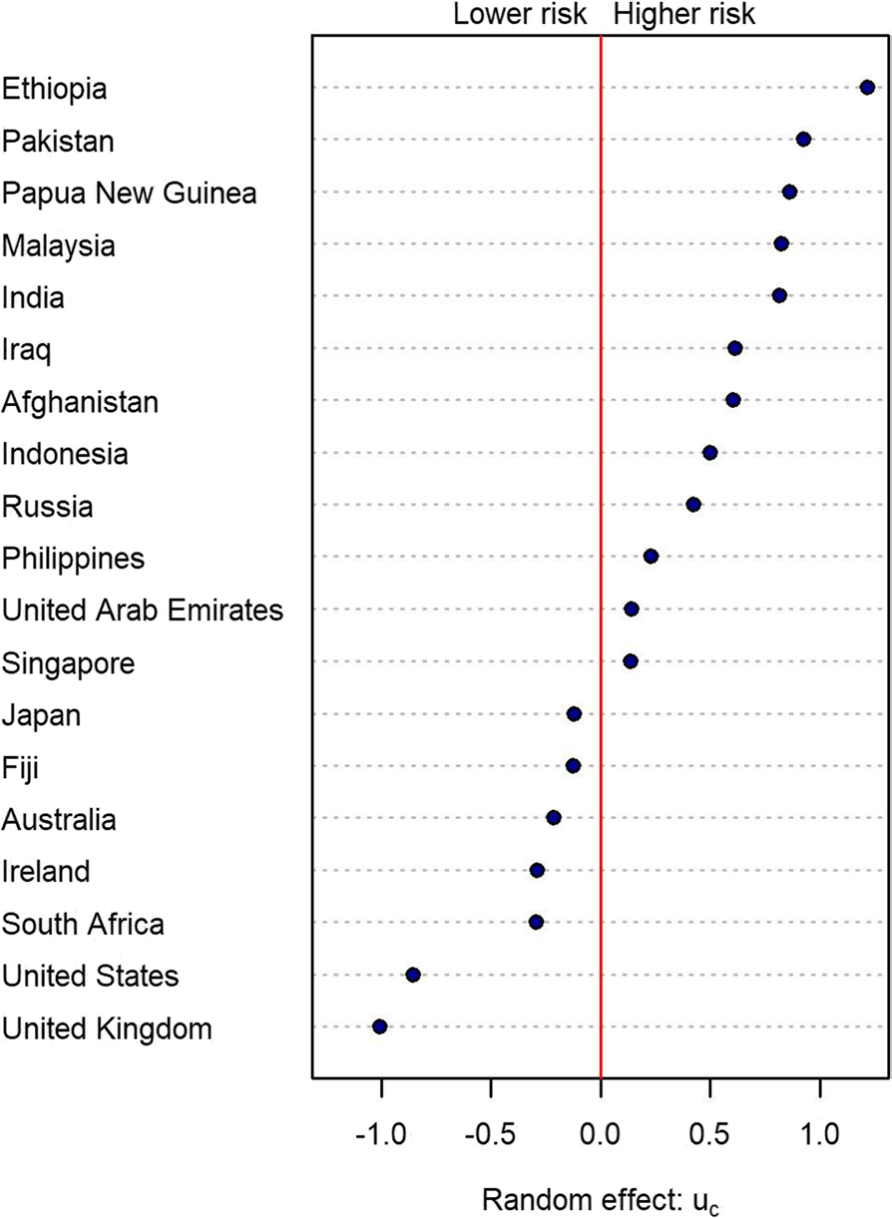
Time-independent random effects $u_c$ for the 19 fully modelled countries. On average, travellers from countries with positive random effects have higher rates of infection than the reported case rate in the general population would predict; travellers from countries with negative random effects have lower rates of infection than the reported case rate would predict. Model fitted to data from 25 January to 22 August 2021

Figure 3 shows the estimated time-independent random effects $\widehat{u}_c$ for the 19 fully modelled countries. On average, over the time period considered, travellers from countries with a positive random effect have a higher risk of infection that would be predicted from the reported case rate $I_{ct}$ in that country, whereas travellers from countries with a negative random effect have a lower risk of infection. The time-dependent random effects $v_{ct}$ represent short-term variations in the infection risk among travellers relative to the long-term average (see Additional file 1: Fig. S5).

The random effects are the mechanism by which the infection rate in arrivals from different countries is modified by factors other than reported cases in the country of origin. As noted already, there is a multitude of such factors. For example, for countries where community transmission has been eliminated or suppressed to very low levels, the majority of reported cases may be in managed isolation facilities, or may be rapidly identified and isolated by contact tracing systems. This could mean that the infection risk in arrivals to New Zealand is lower than reported cases in those countries would suggest. Conversely, the infection risk in arrivals from countries with high levels of unreported infection is likely to be higher than would be predicted based on reported cases alone. This might be viewed as an argument for including number of tests per capita or test positivity rate as predictors in the model. However, we found that including these variables and others such as death rates and vaccination rates did not improve model performance (see Additional file 1: Supplementary methods).

Due to lags between infection and reporting, countries where the epidemic is rapidly growing might be expected to pose a higher risk than countries where it is declining. This could be accounted for by including estimates of the effective reproduction number $R_\text {eff}$ as a predictor in the model. However, again we found that this did not improve model performance beyond the simple extrapolation of epidemic growth/decay that we used to forecast reported cases (see Additional file 1: Sec. S2.8).

Also of importance are any factors that mean international travellers from the country differ in their risk profile from the average resident in the source country. These include travel restrictions and interventions designed to reduce risk (e.g. pre-departure tests, symptom screening, vaccination requirements) and also individual traveller characteristics (e.g. place of residence within the source country, ability to isolate from risk). Relationships between these variables and infection risk are likely to be time-dependent, highly correlated and are thus difficult to estimate separately from one another.

The random effects model absorbs all of these country-specific and time-varying effects into measures which learn the degree of autocorrelation and the magnitude of variation of differences between risk of infection in arrivals and reported cases per capita in the country of origin.

**Fig. 4:**
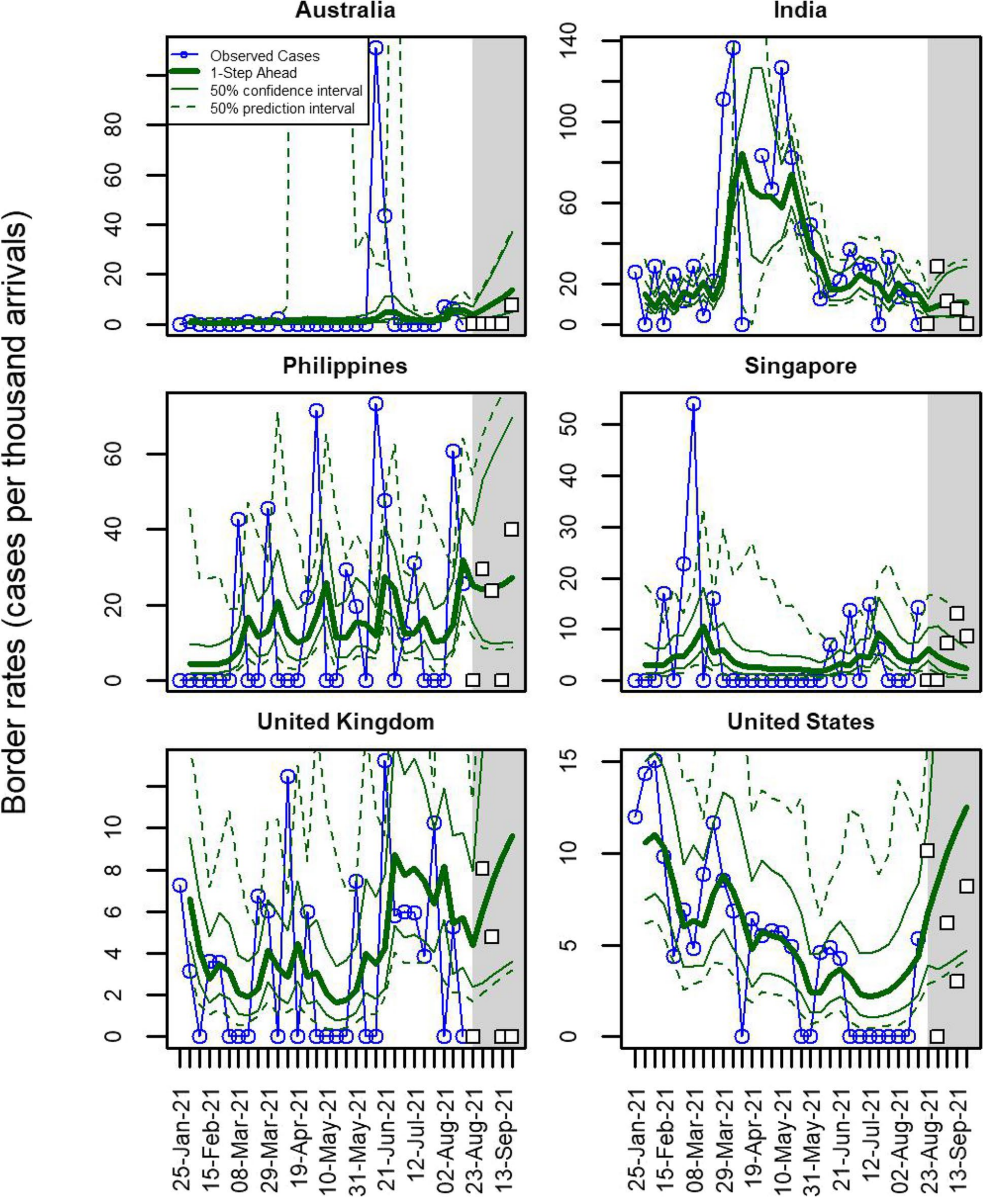
One step ahead predictions for infection rates among arriving cases for the 6 fully modelled countries with the highest numbers of arrivals. Observed rates are shown in blue, the thick green lines show the one step ahead predictions with 50% confidence intervals (thin green lines) and 50% prediction intervals (dashed green lines). Model fitted to data from 25 January to 22 August 2021. Shaded grey region shows a 5-week forecast period alongside actual data for the first 5 weeks of the forecast period (white squares)

One step ahead predictions provide a way to assess the model’s goodness-of-fit. At each time step we use the current covariates and parameter estimates, but forecast the time-dependent random effect forwards from the previous week (shown in Fig. 4 for the same six countries as Fig. 2. Results for all 19 modelled countries are shown in Additional file 1: Fig. S6). As is typical of one step ahead predictions, they appear to slightly lag the true data, with each observation predicting forwards a modified version of its own value.

**Fig. 5:**
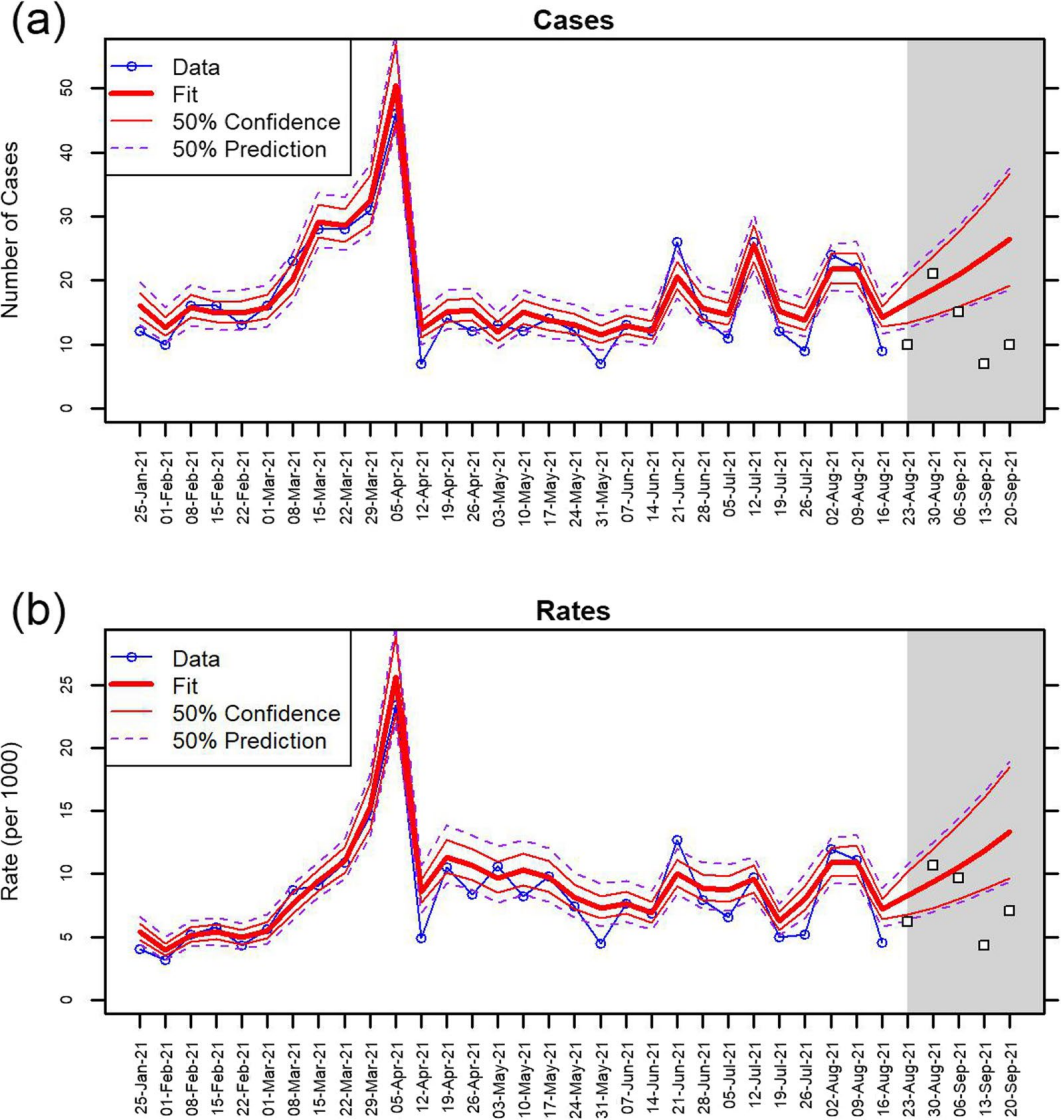
**a** Number of infected arrivals and **b** infection rate per 1000 arrivals aggregated over all countries, showing the model’s central estimate (bold red line), 50% confidence interval (thin red lines) and 50% prediction (dashed purple lines). Model fitted to data from 25 January to 22 August 2021. Shaded grey region shows a 12-week forecast period alongside actual data for the first 5 weeks of the forecast period (white squares)

The country-level estimates can be aggregated over all countries to create estimates of the total numbers of cases that are expected at the border each week (Fig. 5). These estimates include all fully modelled countries as well as those with low and zero case counts.

### Risk classification and aggregate risk estimates

Using the model estimates for the expected numbers of cases and the expected infection rates, we assigned each country to a risk category using specified thresholds. We used the upper bound of the 50% confidence interval for the infection rate to classify countries into four groups using cut-points of 3, 8 and 20 cases per thousand arrivals.

**Fig. 6:**
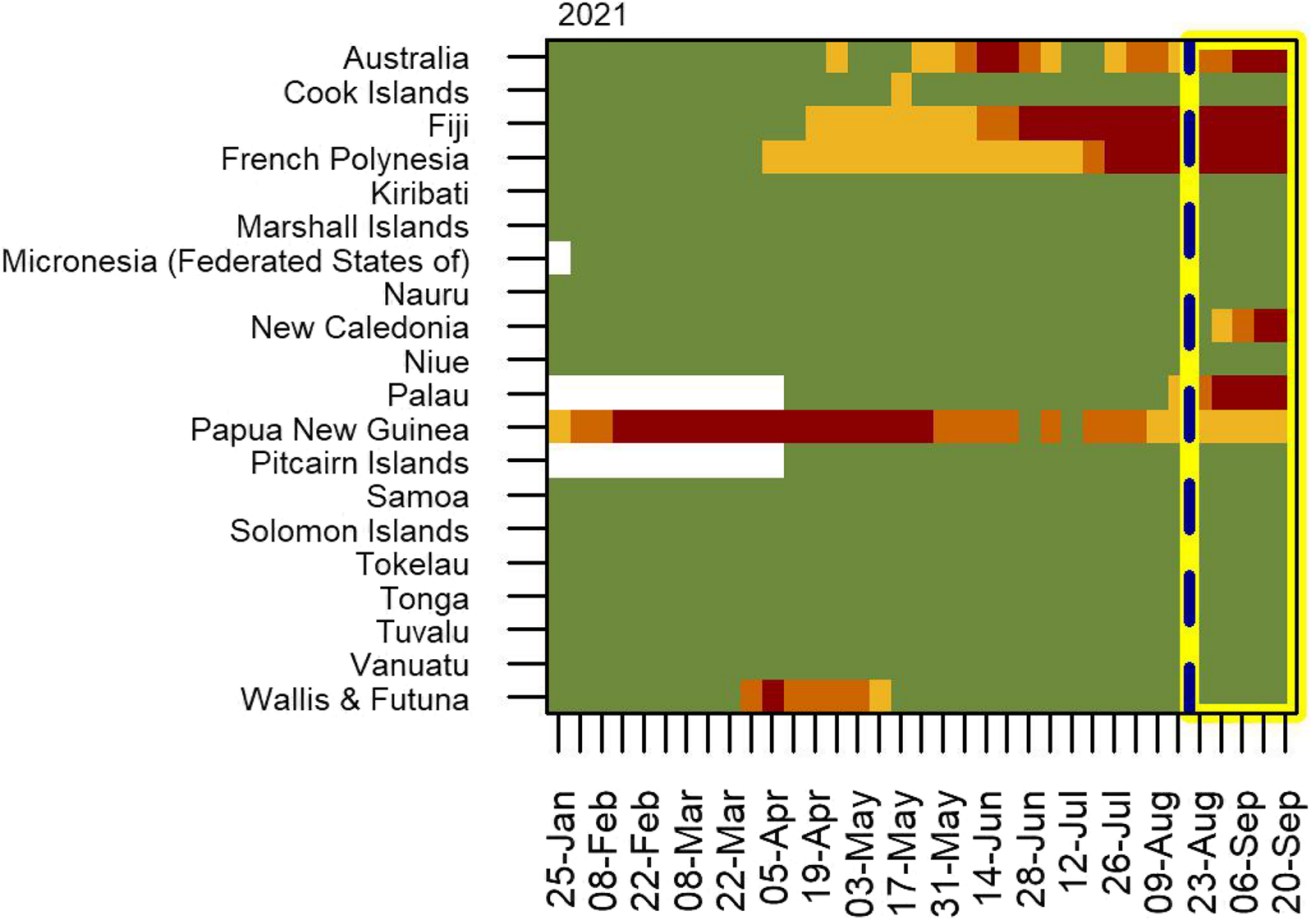
Risk classification for countries in Oceania in 2021. Countries are classified into four risk categories (lowest risk = green, highest risk = dark red, insufficient data = white) using the upper bound of the 50% confidence interval for the infection rate with cut-points of 3, 8 and 20 cases per thousand arrivals. Forecasts are in the area bounded by the yellow box at rights

**Fig. 7:**
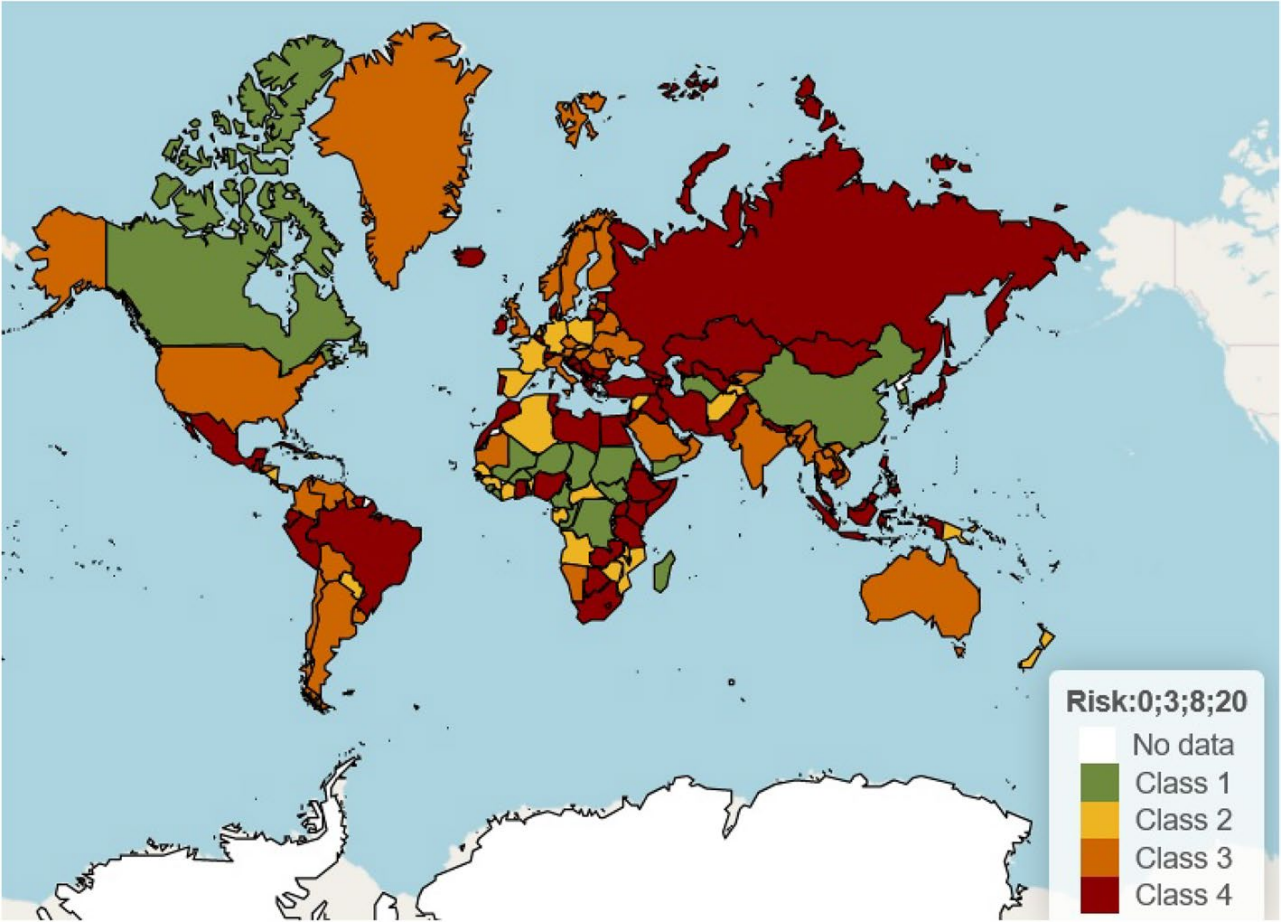
Map of border risk for arrivals from countries around the world for the week starting 23 August 2021. Estimates based on observed data up to 22 August 2021. Countries are classified into four risk categories (lowest risk = green, highest risk = dark red) using the upper bound of the 50% confidence interval for the infection rate with cut-points of 3, 8 and 20 cases per thousand arrivals

Figure 6 shows the changing classification over time for modelled countries in Oceania during 2021. In the forecast period (from 23 August onwards), the risk categorisations of New Caledonia and Palau were expected to increase from the lowest category (green) to the highest (dark red) in the near future. Fiji, French Polynesia, and (to lesser extent) Australia and Papua New Guinea were classified as current and continuing risks. A world risk map is shown in Fig. 7 for the week starting 23 August 2021, estimated using observed data up to 22 August 2021 (i.e. predictions are shown for the first week of the forecast period) using the same four risk categories.

### Model fit and forecast performance

We now consider aspects of model fit and the performance of the forecasts using the methods outlined under assessing model fit in the ‘Methods’ section above.

Firstly, the values of the mean absolute deviation (MAD) and AIC are shown in Table 2 for the main analysis data set used in this section: smaller values of both criteria indicate a better fit, and both support the inclusion of both a country level random effect and the autoregressive component.

**Table 2:** Goodness of fit statistics (AIC and one step ahead mean absolute deviation, MAD) for the fitted model with and without random effects

Model	AIC	MAD
No random effects	1815	1.022
$+$ country level RE	1328	0.780
$+$ AR(1) correlation	1114	0.688

**Fig. 8:**
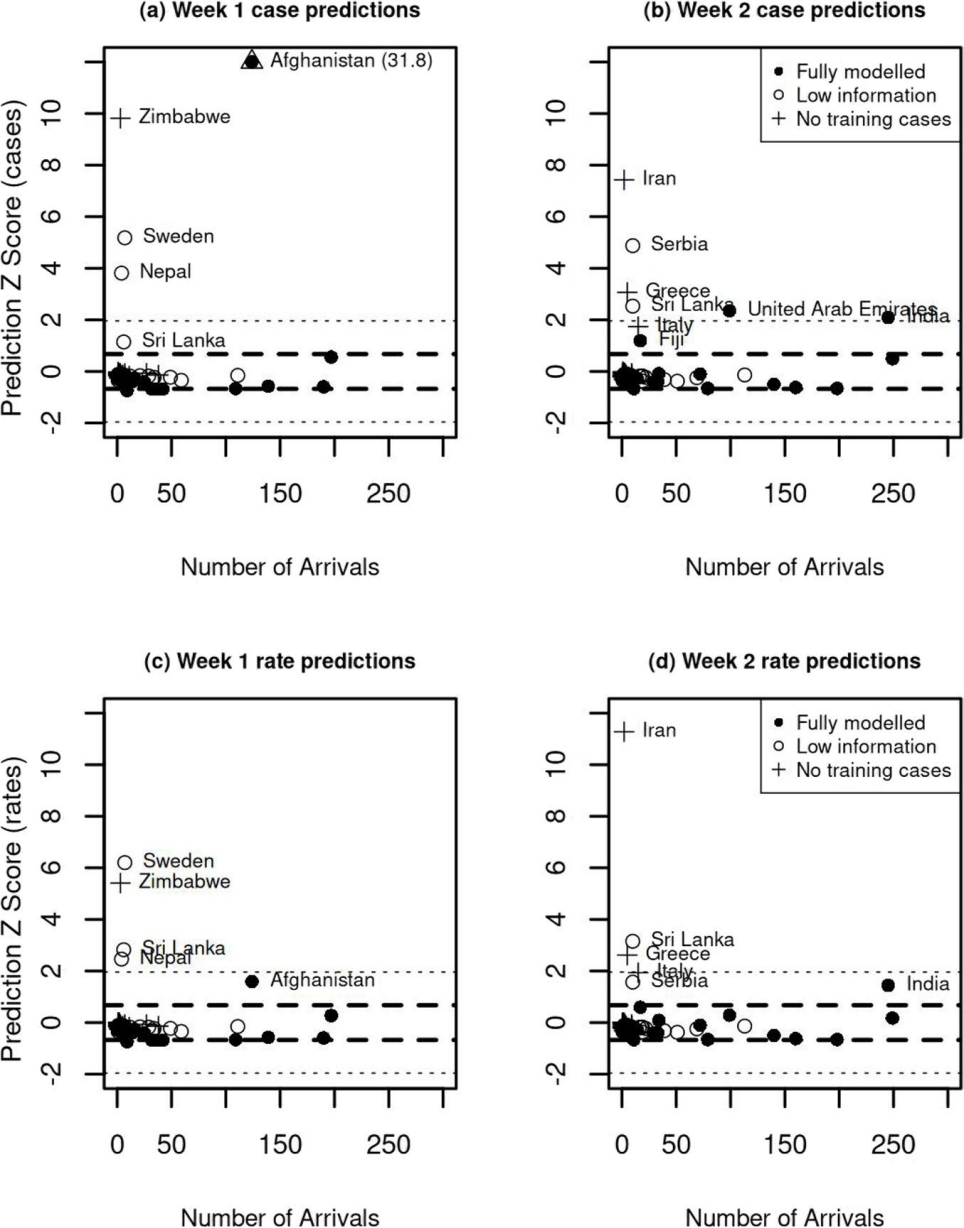
*Z*-scores of case and rate predictions for the first two forecast weeks. Standard 50% and 95% reference ranges are shown by dashed and dotted lines. (In panel **a** Afghanistan should be plotted at $Z=31.8$, but is shown at a lower value for readability)

Secondly, we can assess forecast performance using the *Z*-scores for cases and rates defined in (3). These scores are plotted against the actual numbers of arrivals for the first two forecast weeks in Fig. 8, together with the approximate 50% and 95% ranges within which the scores are expected to lie. Large positive *Z*-scores indicate that the observed cases/rates are higher than the expected values.

Each country for which there was at least one arrival is plotted using a symbol to indicate whether it was one of the 19 fully modelled countries, one of the 44 countries with low information (at least one case in the model training interval), or one of the 161 countries for which there were no cases in the model training interval. We have plotted all of the data on common axes, but this means that the case *Z*-score for Afghanistan in Fig. 8a is plotted at the top of the panel, whereas its actual value is $Z_{\text {cases}}=31.8$.

We note that almost all countries lie within the expected ranges of *Z*-score values for both rates and cases. With the exception of the *Z* case score for Afghanistan in week 1 (Fig. 8a), all countries with strong excesses in *Z-*scores occur for countries with small numbers of arrivals, and none of them are in the fully modelled set of countries. Afghanistan is noteworthy due to having a highly volatile number of arrivals in week 1: 6.7 were forecast but there were 124 actual arrivals.

Among the *Z*-scores for rates in week 1 (Fig. 8c) the scores for all four of the countries outside the 95% range are caused by a single case among a very small number of arrivals. The *Z*-score for the infection rate for Afghanistan is within the expected range of values indicating consistency with the model.

In week 2, the picture is similar to that in week 1, with excesses caused by small numbers of cases among small numbers of arrivals.

Finally, we can compare the forecast and actual risk aggregated over all countries. Figure 5 shows that within the first 3 weeks the forecast rates and case counts match very well, with the actual values sitting within, or close to, the 50% prediction intervals.

We noted in the ‘Methods’ section above that we have not explicitly included any quantitative effect of uncertainties in the forecasting of arrivals ($N_{ct}$) and in-country incidence ($I_{ct}$) in the confidence and prediction intervals associated with our forecasts. To recap briefly, we forecast the numbers of arrivals at a constant rate at the average of the previous 3 weeks of arrival numbers, and forecast incidence using a log linear fit through the last 3 weeks of in-country incidence. (Additional file 1: Figs. S7 and S8 display the forecasts for the 19 fully modelled countries.) We have found that these forecast estimates do in general lead to reliable estimates of the rates of border cases, as can be seen in the results shown in Fig. 8: most *Z*-scores being close to zero and within the expected ranges.

However, where there are sudden changes in the patterns of arrivals or changes in the trajectory of the pandemic in a country then our forecasts will be incorrect and our risk estimates likewise affected. These changes cannot be learned from within the model, and thus cannot be anticipated. The observed excess of actual cases compared to predicated cases for Afghanistan in week 1 is an example of severely underestimated arrivals. We can mitigate some uncertainty in arrivals by using future MIQ bookings, and also estimate the effects on arrivals of changes in policy settings, such as the opening or closing of a quarantine free travel arrangement. (Such an arrangement with Australia ended shortly before the forecast period, and led to an overestimate of expected arrivals and hence of cases from Australia in the results presented here: c.f. Fig. 2.)

Without full models of the pandemic within every country, sharp changes in the trajectory of the pandemic (such as the peaking of cases or a new outbreak) lead to unforseeable changes in the estimated incidence rate. The effects of these changes will be learned by the model in future forecast periods. For policy makers, the reports made by international health monitors, state health agencies and the media will always need to be used to inform overall risk assessments, in addition to the estimates a model such as ours can provide.

## Discussion

The risk assessment model presented here is a robust and practical tool for forecasting the infection risk in border arrivals. It uses readily available data: reported cases in the source countries, together with counts of arrivals and cases by country of origin. These data were collected routinely in New Zealand during the first 2 years of the COVID-19 pandemic, when all international arrivals were PCR tested at least twice. Other countries that imposed strict border testing requirements may also have comparable datasets [16].

The forecasts produced by the model are in the form of expected rates and counts of infected arrivals by country, and are accompanied with suitable measures of uncertainty. These model outputs could be considered by policymakers alongside other information (e.g. effective reproduction number estimates, death rates, testing rates) to support informed, evidence-based decisions about risk posed by travellers arriving from different countries.

Public health and policy responses to the COVID-19 pandemic, and data collection and reporting standards, have been highly variable. This led us to use as simple a modelling approach as possible, using reported cases in the country of origin as the only covariate, with three lags to measure epidemic trajectory. We used a random effects structure to absorb many of the differences between countries that are impossible to model comprehensively. These include the differing levels of unreported infection among countries, and any systematic differences between the resident population (which generates the reported case statistics) and international travellers, who are likely to be healthier and wealthier. The random effects may also absorb changing risks due to different variants of SARS-CoV-2, and changing levels of population immunity due to vaccination programmes, prior infection, and waning. The random effects structure allowed us to estimate the time-varying difference between reported cases per capita in each country of origin and infection risk at the New Zealand border.

Strengths of our study include that it uses data from routine testing of all international arrivals consistently collected over a period of around 18 months. All arrivals were required to spend 14 days in managed isolation and quarantine and, during this time, were PCR tested at least twice and interviewed daily about symptoms. This means that the number of missed infections is likely to be very low and the risk of travellers being infected after arrival, which could otherwise bias infection risk estimates upwards, is also very low. The dataset includes travellers from a large number of different countries, in contrast to other studies which have focused on travellers from a small selection of countries (e.g. [16]). Model output enables user to define indicative risk categories based on multiple variables in a flexible way. For example, a country or group of countries could be classified as high risk if the estimated probability of infection was above 5% or there was a greater than 25% chance of there being more than 20 infected arrivals in 1-week time period. Risk thresholds can be adjusted upwards or downwards over time to suit the current epidemiological situation and public health aims.

Limitations include that the model does not account for the effect of pre-departure travel measures. The effect of such measures can be modelled [6, 16, 19]. However, their effect is complicated by potential compensatory behaviour (e.g. postponing risky activities like social gatherings until after taking a mandatory pre-departure test), screening of travellers based on symptoms at time of departure, and other testing requirements imposed by airlines or transit countries, as opposed to the destination country.

The dataset we used identifies a traveller’s country of origin via arrival card data. New Zealand residents are asked which country they spent the most time in while overseas. Non-residents are asked which country they last lived in for 12 months or more. In some instances, this may not accurately capture the country or countries in which the traveller’s greatest risk of exposure occurred.

The model does not explicitly account for risk of transmission in transit. Air travellers from different countries share airports and aircraft with one another, and in-transit risks may pose significant risks to individual travellers from low-risk countries as they mix with travellers from higher-risk countries. If travellers from a particular country tend to use the same routes, and mix with passengers from the same set of other countries, then to some extent this in-flight risk is incorporated in the random effects structure as a component of the difference between the travelling population at the resident population from any given country. Beyond this observation, we cannot see a reliable source of data by which we could incorporate in-flight transmission into our model.

As noted above, the outputs of the model should not be used as an automatic risk classification system without considering other factors, such as reports of sudden changes in infection rates or the emergence of a new variant in a source country, and the effect of travel restrictions on individuals and families affected.

Compared to countries with land borders, or those at short sea distances from their neighbours, the geographical isolation of New Zealand puts it in an excellent position to be able to monitor and forecast the risk of international arrivals being infected with SARS-CoV-2 or potentially other pathogens. Models created in other countries generally focus on the risks that imported cases pose to the epidemic within that country (e.g. [11, 29, 30]), and this question has also received consideration in New Zealand (e.g. [10]). However, specific models designed for the quantitative assessment of risks in real-time are rare. Lee et al. [31], for example, created a country risk model for arrivals to South Korea where the arrival risk was simply proportional to monthly reported cases in the country of origin.

Other modelling approaches exist as well, with Wang et al. [32] aggregating risk along the route taken by an arriving ship based on the current case numbers and rates of change at the ports visited. Zhang et al. [33] used a model incorporating the connectivity of the international air travel network to assess border risk at provincial level in China. Quilty et al. [19] investigated the aggregate risk of arrivals from all origins, calibrated using flight data, with an interest in optimal testing policies for international arrivals. Their study used the methods of Russell et al. [9, 21] to account for unreported infections in the country of origin by using death rates rather than reported cases as an indicator of prevalence.

The International Civil Aviation Organisation (ICAO) has published suggestions for creating a country risk classification [34]. They proposed a four level classification system based on the percentage of non-immune persons, the 7-day prevalence, the test positivity rate and the testing rate. Classification is based on a set of thresholds of these measures. Where countries have imposed differential treatment of arrivals from source countries, as opposed to treating all arrivals in the same way, it is likely that some version of rules based on these measures has been applied.

Our methodology provides a finer calibration of the border risk posed by arrivals from different countries by incorporating the additional information gained from observing recent arrivals in real time. Our methodology does not rely on assumptions such as a fixed case-fatality ratio nor a fixed level of case ascertainment. Such assumptions need constant revision when surveillance effort or reporting practices change, when new variants emerge, when new treatments are made available, and as population immunity changes over time. Such changes affect border risk, but cannot be easily or separately estimated. Since our method is directly calibrated by actual arrivals, we estimate the combined effect of these factors in the random effects structure.

Our approach is of course at risk of missing rapid changes in source countries since there is typically a lag from infection to reporting, and it takes time for the model to adjust to abrupt shifts in the level of risk.

## Conclusions

We have developed a forecasting approach suitable for assisting border control decisions to control the risk of infected individuals arriving. The model differentiates between countries, using real time measurements of in country infection rates together with recent actual rates of infection among border arrivals to forecast risk for each country. The temporal random effects structure allows the model to adapt to sudden changes in each source country.

We reiterate that border risk assessment decisions need to rely on a wide range of data sources and considerations, of which our model is only one component. We agree with the advice given in the Interational Civil Aviation Organisations report that ‘although data-driven decision making is encouraged, the current scenario may require a qualitative approach, as validated data and information is incomplete’ [34].

## Supplementary Information

**Additional file 1.** One additional file accompanies this paper: ‘Additional file 1 for: Estimating the risk of SARS-CoV-2 infection in New Zealand border arrivals’. It contains further technical details of the data, the statistical model, and the estimation and forecasting procedures. It also contains details of the model selection procedure and results, as well as additional figures showing the fitted model and forecasts.

## Data Availability

Anonymised unit record case data was received from the Institute of Environmental Science and Research under a data sharing agreement. The New Zealand Ministry of Health retains all control of the case and arrivals data. Data on reported cases and other covariates for countries of origin was retrieved from the open access site Our World in Data https://ourworldindata.org, and were originally supplied by Johns Hopkins University. R code used to fit the model is available at https://gitlab.com/arnoldri/nzarrivalrisk [28].

## Abbreviations

AIC: Akaike information criterion
COVID-19: Coronavirus disease caused by SARS-CoV-2
ESR: Institute of Environmental and Scientific Research
ICAO: International Civial Aviation Organisation
MAD: Mean absolute deviation
MIQ: Managed isolation and quarantine
PCR: Polymerase chain reaction
RE: Random effect
SARS-CoV-2: Severe acute respiratory syndrome-related coronavirus
TMB: Template Model Builder

## References

[1] Hale T, Angrist N, Goldszmidt R, Kira B, Petherick A, Phillips T, et al. A global panel database of pandemic policies (Oxford COVID-19 Government Response Tracker). Nat Hum Behav. 2021;5(4):529–38. 33686204 10.1038/s41562-021-01079-8

[2] Grout L, Katar A, Ait Ouakrim D, Summers JA, Kvalsvig A, Baker MG, et al. Failures of quarantine systems for preventing COVID-19 outbreaks in Australia and New Zealand. Med J Aust. 2021;215(7):320–4. 34472122 10.5694/mja2.51240 PMC8661623

[3] Mendelson M, Venter F, Moshabela M, Gray G, Blumberg L, de Oliveira T, et al. The political theatre of the UK’s travel ban on South Africa. Lancet. 2021;398(10318):2211–3. 34871546 10.1016/S0140-6736(21)02752-5 PMC8641956

[4] Steens A, Freiesleben de Blasio B, Veneti L, Gimma A, Edmunds WJ, Van Zandvoort K, et al. Poor self-reported adherence to COVID-19-related quarantine/isolation requests, Norway, April to July 2020. Eurosurveillance. 2020;25(37):2001607. 10.2807/1560-7917.ES.2020.25.37.2001607 PMC7502884 32945254

[5] Eichler N, Thornley C, Swadi T, Devine T, McElnay C, Sherwood J, et al. Transmission of severe acute respiratory syndrome coronavirus 2 during border quarantine and air travel, New Zealand (Aotearoa). Emerg Infect Dis. 2021;27(5):1274. 33734063 10.3201/eid2705.210514 PMC8084504

[6] Steyn N, Lustig A, Hendy SC, Binny RN, Plank MJ. Effect of vaccination, border testing, and quarantine requirements on the risk of COVID-19 in New Zealand: A modelling study. Infect Dis Model. 2022;7(1):184–98. 34977439 10.1016/j.idm.2021.12.006 PMC8712670

[7] Jackson C, Habibi R, Forman L, Silva DS, Smith MJ. Between rules and resistance: moving public health emergency responses beyond fear, racism and greed. BMJ Global Health. 2022;7(12). 10.1136/bmjgh-2022-009945. 10.1136/bmjgh-2022-009945 PMC9723907 36593643

[8] Clifford S, Quilty BJ, Russell TW, Liu Y, Chan YWD, Pearson CA, et al. Strategies to reduce the risk of SARS-CoV-2 importation from international travellers: modelling estimations for the United Kingdom, July 2020. Eurosurveillance. 2021;26(39):2001440. 34596018 10.2807/1560-7917.ES.2021.26.39.2001440 PMC8485583

[9] Russell TW, Wu JT, Clifford S, Edmunds WJ, Kucharski AJ, Jit M. Effect of internationally imported cases on internal spread of COVID-19: a mathematical modelling study. Lancet Public Health. 2021;6:e12-20. 10.1016/S2468-2667(20)30263-2. 33301722 10.1016/S2468-2667(20)30263-2 PMC7801817

[10] Steyn N, Plank MJ, James A, Binny RN, Hendy SC, Lustig A. Managing the risk of a COVID-19 outbreak from border arrivals. J R Soc Interface. 2021;18:20210063. 10.1098/rsif.2021.0063. 33878278 10.1098/rsif.2021.0063 PMC8086931

[11] Hurford A, Rahman P, Loredo-Osti JC. Modelling the impact of travel restrictions on COVID-19 cases in Newfoundland and Labrador. R Soc Open Sci. 2021;8(6):202266. 34150314 10.1098/rsos.202266 PMC8206704

[12] Zachreson C, Shearer FM, Price DJ, Lydeamore MJ, McVernon J, McCaw J, et al. COVID-19 in low-tolerance border quarantine systems: Impact of the Delta variant of SARS-CoV-2. Sci Adv. 2022;8(14):eabm3624. 35394833 10.1126/sciadv.abm3624 PMC8993115

[13] Kucharski AJ, Jit M, Logan JG, Cotten M, Clifford S, Quilty BJ, et al. Travel measures in the SARS-CoV-2 variant era need clear objectives. Lancet. 2022;399(10333):1367–9. 35247312 10.1016/S0140-6736(22)00366-X PMC8890754

[14] Baker MG, Wilson N, Anglemyer A. Successful elimination of Covid-19 transmission in New Zealand. N Engl J Med. 2020;383(8):e56. 32767891 10.1056/NEJMc2025203 PMC7449141

[15] Plank MJ, Hendy SC, Binny RN, Vattiato G, Lustig A, Maclaren OJ. Using mechanistic model-based inference to understand and project epidemic dynamics with time-varying contact and vaccination rates. Sci Rep. 2022;12(1):20451. 36443439 10.1038/s41598-022-25018-3 PMC9702885

[16] Kucharski A, Chung K, Aubry M, Teiti I, Teissier A, Richard V, et al. Real-time surveillance of international SARS-CoV-2 prevalence using systematic traveller arrival screening. PLoS Med. 2023;20(9):e1004283. 10.1371/journal.pmed.1004283. 37683046 10.1371/journal.pmed.1004283 PMC10516411

[17] Pouwels KB, House T, Pritchard E, Robotham JV, Birrell PJ, Gelman A, et al. Community prevalence of SARS-CoV-2 in England from April to November, 2020: results from the ONS Coronavirus Infection Survey. Lancet Public Health. 2021;6(1):e30–8. 33308423 10.1016/S2468-2667(20)30282-6 PMC7786000

[18] Riley S, Atchison C, Ashby D, Donnelly CA, Barclay W, Cooke GS, et al. Real-time Assessment of Community Transmission (REACT) of SARS-CoV-2 virus: study protocol. Wellcome Open Res. 2020;5:200. 10.12688/wellcomeopenres.16228.1 PMC8095190 33997297

[19] Quilty BJ, Russell TW, Clifford S, Flasche S, Pickering S, Neil SJ, et al. Quarantine and testing strategies to reduce transmission risk from imported SARS-CoV-2 infections: a global modelling study. medRxiv. 2021. 10.1101/2021.06.11.21258735.

[20] Strohmeier M, Olive X, Lübbe J, Schäfer M, Lenders V. Crowdsourced air traffic data from the OpenSky Network 2019–2020. Earth Syst Sci Data. 2021;13(2):357–66.

[21] Russell TW, Golding N, Hellewell J, Abbott S, Wright L, Pearson CAB, et al. Reconstructing the early global dynamics of under-ascertained COVID-19 cases and infections. BMC Med. 2020;18:332. 33087179 10.1186/s12916-020-01790-9 PMC7577796

[22] Global Change Data Lab. Our World in Data - COVID19 data set. https://github.com/owid/covid-19-data/raw/master/public/data/. Accessed 17 May 2022.

[23] Arnold R, Binny RN, Lumley T, Lustig A, Parry M, Plank M. Estimating COVID19 Border Arrival Risk in New Zealand. Covid-19 Modelling Aotearoa. 2022. https://www.covid19modelling.ac.nz/estimating-covid-19-border-arrival-risk-in-aotearoa-new-zealand/. Accessed 7 Mar 2024.

[24] Institute of Environmental and Scientific Research (ESR). EpiSurv database. 2024. https://episurv.esr.cri.nz/. Accessed 7 Mar 2024.

[25] Funk S, Endo A, Robert A, Gruson H, Munday J, Sherratt K, et al. Epiforecasts: Real-time modelling and forecasting of infectious disease dynamics. https://epiforecasts.io/. Accessed 17 May 2022.

[26] Brooks ME, Kristensen K, van Benthem KJ, Magnusson A, Berg CW, Nielsen A, et al. glmmTMB Balances Speed and Flexibility Among Packages for Zero-inflated Generalized Linear Mixed Modeling. R Journal. 2017;9(2):378–400. https://journal.r-project.org/archive/2017/RJ-2017-066/index.html.

[27] Kristensen K, Nielsen A, Berg CW, Skaug H, Bell BM. TMB: Automatic Differentiation and Laplace Approximation. J Stat Softw. 2016;70(5):1–21. 10.18637/jss.v070.i05.

[28] Arnold R, Binny RN, Lumley T, Lustig A, Parry M, Plank MJ. NZ Arrival Risk. 2024. R code implementing the NZ arrival risk forecasting model. https://gitlab.com/arnoldri/nzarrivalrisk. Accessed 16 Oct 2023.

[29] Koutsellis T, Nikas A. A predictive model and country risk assessment for COVID-19: An application of the Limited Failure Population concept. Chaos Solitons Fractals. 2020;140:110240. 32863614 10.1016/j.chaos.2020.110240 PMC7444907

[30] Zhu Z, Weber E, Strohsal T, Serhan D. Sustainable border control policy in the COVID-19 pandemic: A math modeling study. Travel Med Infect Dis. 2021;41:102044. 10.1016/j.tmaid.2021.102044. 33838318 10.1016/j.tmaid.2021.102044 PMC8025603

[31] Lee H, Kim Y, Kim E, Lee S. Risk Assessment of Importation and Local Transmission of COVID-19 in South Korea: Statistical Modeling Approach. JMIR Public Health Surveill. 2021;7(6):e26784. 10.2196/26784. 33819165 10.2196/26784 PMC8171290

[32] Wang Z, Yao M, Meng C, Claramunt C. Risk Assessment of the Overseas Imported COVID-19 of Ocean-Going Ships Based on AIS and Infection Data. ISPRS Int J Geo-Inf. 2020;9(6):351. 10.3390/ijgi9060351.

[33] Zhang L, Yang H, Wang K, Zhan Y, Bian L. Measuring imported case risk of COVID-19 from inbound international flights – A case study on China. J Air Transp Manag. 2020;89:101918. 10.1016/j.jairtraman.2020.101918. 32904487 10.1016/j.jairtraman.2020.101918 PMC7455240

[34] International Civil Aviation Organization. Doc 10152: Manual on COVID19 cross-border risk management. 2021. https://www.icao.int/covid/cart/Documents/10152_manual_3rd_edition.en.pdf. Accessed 17 May 2022.

