# Supplementary material for: Estimating the risk of SARS-CoV-2 infection in New Zealand border arrivals
Source: BMC Glob Public Health. 2024 May 3;2:27. doi: 10.1186/s44263-024-00057-2 (PMC11622930; doi:10.1186/s44263-024-00057-2)
Supplement: Supplementary file 1 — Additional file 1. One additional file accompanies this paper: ‘Additional file 1 for: Estimating the risk of SARS-CoV-2 infection in New Zealand border arrivals’. It contains further technical details of the data, the statistical model, and the estimation and forecasting procedures. It also contains details of the model selection procedure and results, as well as additional figures showing the fitted model and forecasts. [file 44263_2024_57_MOESM1_ESM.pdf]

Additional file 1 for: Estimating the risk of SARS-CoV-2 infection in New Zealand border arrivals

Richard Arnold[1], Rachelle N. Binny[2], Thomas Lumley[3], Audrey Lustig[2], Matthew Parry[4], Michael J. Plank[5]

Version: 8 March 2024

1. School of Mathematics and Statistics, Victoria University of Wellington, New Zealand.

2. Manaaki Whenua - Landcare Research, Lincoln, New Zealand.

3. Department of Statistics, University of Auckland, Auckland, New Zealand.

4. Department of Mathematics and Statistics, University of Otago, Dunedin, New Zealand.

5. School of Mathematics and Statistics, University of Canterbury, Christchurch, New Zealand.

Supplementary data description

- We assume that the data on new arrivals and counts of cases are complete in New Zealand. New Zealand's geographic isolation means it has no land borders, and that unreported arrivals by sea are rare. Arrivals by air are controlled at New Zealand's international airports.
- The information on the country of origin of each arrival is collected in Question 2 of the passenger arrival card completed by all passengers and crew on aeroplanes and ships arriving in New Zealand.

  There are two versions of the question (see Figure S1):

  1. **Question 2a:** 'Answer this question if you live in New Zealand: . . . Which country did you spend the most time in while overseas?'

Table S1: New Zealand border restrictions during the Covid-19 pandemic.

Date	Measures taken	Reference
2 Feb 2020	Travellers leaving from or transitting through China will be refused entry; Foreign travellers subject to increased screening on arrival; NZ citizens and residents must self-isolate for 14 days	[1]
19 Mar 2020	NZ borders closed except to NZ citizens and permanent residents, their partners and children and some health and humanitarian workers	[2]
9 Apr 2020	14 day stay in managed isolation and quarantine (MIQ) becomes mandatory for all arrivals	[3]
3 Nov 2020	Advance MIQ bookings become mandatory for all arrivals	[4]
15 Jan 2021	Negative pre-departure tests required for travellers from the UK and US	[5]
18 Jan 2021	Negative pre-departure tests required for all arrivals excepting those from Australia, Antarctica and some Pacific Island nations; Tests in MIQ at Day 0, 3 and 12	[3]
21 Jan 2021	Quarantine-free travel from the Cook Islands starts	[6]
1 Apr 2021	Widening of criteria for emergency bookings in MIQ	[7]
11–18 Apr 2021	Arrivals from India banned for 2 weeks	[8]
19 Apr–23 Jul 2021	Quarantine-free travel from Australia	[3, 9, 10]
28 Apr 2021	India, Brazil, Papua New Guinea and Pakistan classified as Very High Risk: travel restricted to NZ citizens and families	[11, 12]
15 Aug 2021	Fiji and Indonesia classified as Very High Risk: travel restricted to NZ citizens and families	[13, 12]
1 Nov 2021	Full vaccination a requirement for entry for non-citizens	[14]
Feb–Jul 2022	MIQ requirements progressively phased out	[15]

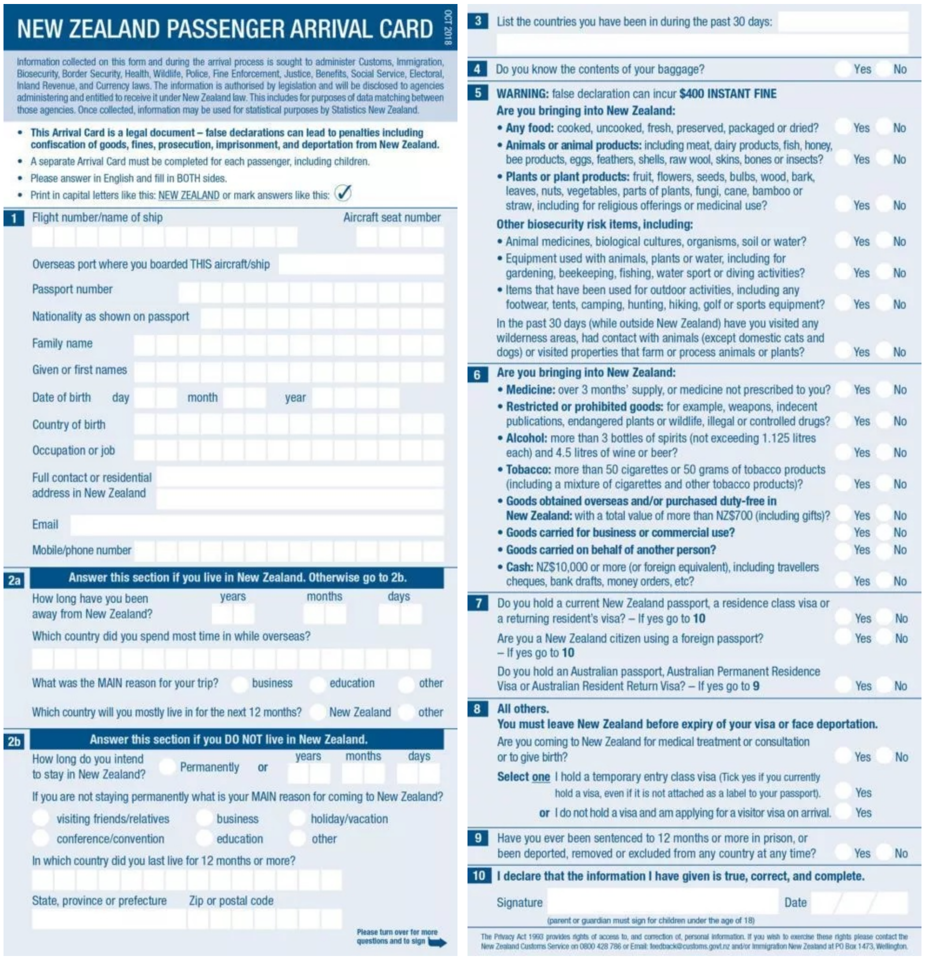

NEW ZEALAND PASSENGER ARRIVAL CARD

OCT 2018

Information collected on this form and during the arrival process is sought to administer Customs, Immigration, Biosecurity, Border Security, Health, Wildlife, Police, Fine Enforcement, Justice, Benefits, Social Service, Electoral, Inland Revenue, and Currency laws. The information is authorised by legislation and will be disclosed to agencies administering and entitled to receive it under New Zealand law. This includes for purposes of data matching between those agencies. Once collected, information may be used for statistical purposes by Statistics New Zealand.

- **This Arrival Card is a legal document – false declarations can lead to penalties including confiscation of goods, fines, prosecution, imprisonment, and deportation from New Zealand.**
- A separate Arrival Card must be completed for each passenger, including children.
- Please answer in English and fill in BOTH sides.
- Print in capital letters like this: NEW ZEALAND or mark answers like this: ✓

1 Flight number/name of ship — Aircraft seat number

Overseas port where you boarded THIS aircraft/ship

Passport number

Nationality as shown on passport

Family name

Given or first names

Date of birth day month year

Country of birth

Occupation or job

Full contact or residential address in New Zealand

Email

Mobile/phone number

2a **Answer this section if you live in New Zealand. Otherwise go to 2b.**

How long have you been away from New Zealand? years months days

Which country did you spend most time in while overseas?

What was the MAIN reason for your trip? business education other

Which country will you mostly live in for the next 12 months? New Zealand other

2b **Answer this section if you DO NOT live in New Zealand.**

How long do you intend to stay in New Zealand? Permanently or years months days

If you are not staying permanently what is your MAIN reason for coming to New Zealand?

visiting friends/relatives business holiday/vacation

conference/convention education other

In which country did you last live for 12 months or more?

State, province or prefecture Zip or postal code

Please turn over for more questions and to sign

3 List the countries you have been in during the past 30 days:

4 Do you know the contents of your baggage? Yes No

5 **WARNING:** false declaration can incur **$400 INSTANT FINE**

Are you bringing into New Zealand:

- **Any food:** cooked, uncooked, fresh, preserved, packaged or dried? Yes No
- **Animals or animal products:** including meat, dairy products, fish, honey, bee products, eggs, feathers, shells, raw wool, skins, bones or insects? Yes No
- **Plants or plant products:** fruit, flowers, seeds, bulbs, wood, bark, leaves, nuts, vegetables, parts of plants, fungi, cane, bamboo or straw, including for religious offerings or medicinal use? Yes No

Other biosecurity risk items, including:

- Animal medicines, biological cultures, organisms, soil or water? Yes No
- Equipment used with animals, plants or water, including for gardening, beekeeping, fishing, water sport or diving activities? Yes No
- Items that have been used for outdoor activities, including any footwear, tents, camping, hunting, hiking, golf or sports equipment? Yes No

In the past 30 days (while outside New Zealand) have you visited any wilderness areas, had contact with animals (except domestic cats and dogs) or visited properties that farm or process animals or plants? Yes No

6 **Are you bringing into New Zealand:**

- **Medicine:** over 3 months' supply, or medicine not prescribed to you? Yes No
- **Restricted or prohibited goods:** for example, weapons, indecent publications, endangered plants or wildlife, illegal or controlled drugs? Yes No
- **Alcohol:** more than 3 bottles of spirits (not exceeding 1.125 litres each) and 4.5 litres of wine or beer? Yes No
- **Tobacco:** more than 50 cigarettes or 50 grams of tobacco products (including a mixture of cigarettes and other tobacco products)? Yes No
- **Goods obtained overseas and/or purchased duty-free in New Zealand:** with a total value of more than NZ$700 (including gifts)? Yes No
- **Goods carried for business or commercial use?** Yes No
- **Goods carried on behalf of another person?** Yes No
- **Cash:** NZ$10,000 or more (or foreign equivalent), including travellers cheques, bank drafts, money orders, etc? Yes No

7 Do you hold a current New Zealand passport, a residence class visa or a returning resident's visa? – If yes go to **10** Yes No

Are you a New Zealand citizen using a foreign passport? – If yes go to **10** Yes No

Do you hold an Australian passport, Australian Permanent Residence Visa or Australian Resident Return Visa? – If yes go to **9** Yes No

8 **All others.**
You must leave New Zealand before expiry of your visa or face deportation.

Are you coming to New Zealand for medical treatment or consultation or to give birth? Yes No

Select one I hold a temporary entry class visa (Tick yes if you currently hold a visa, even if it is not attached as a label to your passport). Yes

or I do not hold a visa and am applying for a visitor visa on arrival. Yes

9 Have you ever been sentenced to 12 months or more in prison, or been deported, removed or excluded from any country at any time? Yes No

10 **I declare that the information I have given is true, correct, and complete.**

Signature — Date

(parent or guardian must sign for children under the age of 18)

The Privacy Act 1993 provides rights of access to, and correction of, personal information. If you wish to exercise these rights please contact the New Zealand Customs Service on 0800 428 786 or Email: feedback@customs.govt.nz and/or Immigration New Zealand at PO Box 1473, Wellington.

Figure S1: New Zealand Passenger Arrival Card

2. **Question 2b:** ‘Answer this question if you DO NOT live in New Zealand: … In which country did you last live for 12 months or more?’

There is also a free text response question **Question 3:** ‘List the countries you have been in during the last 30 days:’ This response is not routinely captured.

The country of origin that is captured in Question 2 may not be the country at which the person was most at risk of infection.

- Arrivals that could not be matched to a standard country name, or where the country did not have corresponding data in the Our World in Data dataset were assigned to ‘Unknown Origin’.

  Between 8 Jun 2020 and 20 Feb 2022 there were 4060 arrivals with unknown origin, out of a total of 207518 arrivals (2.0%).
- Cases are selected from the EpiSurv database if their Status flag has the value ‘Confirmed’ or ‘Probable’, and the Overseas flag is ‘Yes’.
- When an arriving person is found to be infected with COVID19 more information is collected: the three most recently visited countries are captured in the EpiSurv database, along with the departure dates from each country.

  Of these three countries the least recently visited country is selected from the countries that were visited within the previous 14 days.

  Inevitably there are inaccurate dates (e.g. departures taking place before arrivals) and missing data on recently visited countries. Between 8 Jun 2020 and 20 Feb 2022 there were 2 cases with unknown origin, out of a total of 3047 cases arriving at the border (0.1%).
- There is a potential mismatch between the reported origin of cases (from EpiSurv) and the counts of arrivals by country (from the arrival card). For example, there were 10 cases recorded as being from Ukraine reported on 26 January 2021, but only 2 people classified as having arrived from the Ukraine that day.

  Prior to 1 April 2021 the arrival card country is used in the denominator, and the EpiSurv country is used in the numerator.
- All analyses from 1 April 2021 onwards use the Arrival Card country if available.

  If that is not available then the last known port along the passenger’s route is used. If that is not available **and** if the arrival is a case, then the country of origin reported in the EpiSurv database is used (or the ‘Travelled from’ country if that is not available).

This consistency of approach minimises any numerator/denominator bias, although it likely does introduce some misclassification, since the EpiSurv country is more likely to be correct.

During the period 1 April 2021-21 November 2021 there were 60779 arrivals and 486 cases.

Among the 60293 non-cases, there were 695 (1.2%) with no Arrival Card country, similar to the rate (7/486=1.4%) among the cases.

Among the 486 cases:

  - 3 (0.6%) have neither an Arrival Card nor an EpiSurv country;
  - 4 (0.8%) have an EpiSurv country only;
  - 39 (2.5%) have an Arrival Card country only;
  - 68 (14%) have both countries, but they don't match
  - 372 (77%) have both countries and they do match

The 68 cases with non-matching EpiSurv and Arrival Card countries record 26 different EpiSurv countries and 30 different Arrival Card countries. These are all mostly small numbers (1-3 per country), but the largest EpiSurv counts are from Russia (7), the USA (7) and the UK (15). These 29 cases are distributed among 10 different Arrival Card countries, with small numbers from each with the notable exception of 11 cases with the UK as their EpiSurv country, but Singapore as their Arrival Card country.

We can expect over-attribution of cases to the United Arab Emirates, the United States, Qatar and Singapore, which account for the most frequent routes by which travellers come to New Zealand. The consequent under-attribution of cases to the genuine source countries is a small effect for most source countries, given that we have seen very small counts (1-3) missing from a large number of countries (26).

- In situations where the number of cases from a country exceeds the number of arrivals from that country on a particular day, we exclude **ALL** of those cases from the model.
- Inflight infection is not accounted for in the model. However, with the highly transmissible Omicron variant this is more likely to be an issue. Where inflight transmission is known to have occurred, these cases could either be (a) deleted from the case counts (in order not to underestimate this risk of border cases), or (b) reclassified, either at random or proportionately, across a set of countries.
- Data are aggregated to weekly totals (Monday-Sunday) before modelling. This reduces

the proportion of counts that are very small, as well as eliminating any effect of irregular reporting during the various days of the week.

Weeks are numbered in some output: the Monday of Week 1 is 13 January 2020. If the final week has 5 or 6 days we scale the number of cases and arrivals so that the counts are equivalent to a weekly total. If the final week has fewer than 5 days of data all the data for that week are excluded.

We thus have one record of data for each country for each week.

- The Our World in Data dataset contains occasional negative counts of cases, deaths or tests. These are corrections to earlier overcounting. These negative counts are all set to zero, with no other adjustments made.

Supplementary methods

Statistical model

For country c in time period t we use the following notation:

- I_{ct} = reported per capita cases in country c (referred to as the case rate)
- N_{ct} = total number of passengers arriving at the New Zealand border
- Y_{ct} = number of cases arriving at the New Zealand border

All data are aggregated to weekly periods.

We then model the number of arriving cases Y_{ct} as a binomial count model,

$$Y_{ct} \sim \text{Binomial}(N_{ct}, \mu_{ct}) \tag{S1}$$

where

$$\begin{aligned} \eta_{ct} = \text{logit}\mu_{ct} &= \alpha + \sum_{k=k_1}^{k_2} \beta_k \text{logit}\left(\delta + I_{c,t-k}\right) + u_c + v_{ct} \\ u_c &\overset{\text{iid}}{\sim} N(0, \sigma_u^2) \\ v_{ct} &= \rho v_{c,t-1} + \varepsilon_{ct} \\ \varepsilon_{ct} &\overset{\text{iid}}{\sim} N(0, \sigma_e^2) \end{aligned} \tag{S2}$$

and $\delta = 10^{-7}$ is a small offset to case rate observations which allows for situations where the reported case rate I_{ct} is 0. We used minimum lag $k_1 = 0$ and maximum lag $k_2 = 2$.

We use logits to transform proportions and logs to transform non-negative quantities onto the unrestricted linear predictor scale. Since the case rates are in general low ($< 1\%$), this specification encodes the approximate proportionality:

$$\mu_{ct} \propto \prod_{k=k_1}^{k_2} (I_{c,t-k})^{\beta_k} \tag{S3}$$

The random effects structure assigns a country level random effect u_c to country c to account for arrivals from that country differing in risk from the risk level suggested by the reported case rate I_{ct}. The autoregressive AR(1) error structure v_{ct} allows this country level effect to change over time, but with temporal correlation.

The full set of parameters of the model is thus

- α: intercept
- $\beta_{k_1}, \ldots, \beta_{k_2}$: parameters associated with the logit of lagged case rates $I_{t-k_1}, \ldots, I_{t-k_2}$
- ρ: AR(1) correlation parameter
- σ_u^2: variance of country level random effects
- σ_e^2: one step variance of time varying AR(1) random effects

Estimation

The model is fit using the R package `glmmTMB` [16] which implements in R the TMB package [17] for use with generalised linear mixed effects models. We restrict the data to be modelled to data in a fixed window, the most recent 30 weeks, to allow for long term changes in the pandemic.

Given data on cases, arrivals, and case rates in country of origin (Y_{ct}, N_{ct}, I_{ct}) at the n weekly time points $(t_1, \ldots, t_n)$ and countries $c = 1, \ldots, C$ the TMB estimation function returns:

- Parameter estimates $\widehat{\boldsymbol{\beta}}$, $\widehat{\rho}$, $\widehat{\sigma}_u^2$ and $\widehat{\sigma}_e^2$;
- Variance-covariance of $\widehat{\boldsymbol{\beta}}$ estimates: $\widehat{V}_\beta$;
- Estimated country level random effects $\widehat{u}_c$;
- Estimated time dependent random effects $\widehat{v}_{ct}$ for each country at the observed data time points $(t_1, \ldots, t_n)$;

- Fitted values and variance on the logit scale

$$\begin{aligned} \widehat{\eta}_{ct} &= \mathbf{I}_{ct}^T \widehat{\boldsymbol{\beta}} + \widehat{u}_c + \widehat{v}_{ct} \quad \text{(S4)} \\ \text{Var}[\widehat{\eta}_{ct}] &= \widehat{S}_{ct}^2 \quad \text{(S5)} \end{aligned}$$

i.e. $\widehat{S}_{ct}$ is the standard error of the estimate of η_{ct};

- Fitted values and variance on the observation scale

$$\begin{aligned} \widehat{Y}_{ct} = N_{ct}\widehat{\mu}_{ct} &= N_{ct}\text{expit}(\mathbf{I}_{ct}^T \widehat{\boldsymbol{\beta}} + \widehat{u}_c + \widehat{v}_{ct}) \quad \text{(S6)} \\ \text{Var}[\widehat{Y}_{ct}] &= N_{ct}^2 \widehat{S}_{ct}^2 \, \widehat{\mu}_{ct}^2 (1-\widehat{\mu}_{ct})^2 \quad \text{(S7)} \end{aligned}$$

Confidence intervals for η_{ct}, μ_{ct} and $\widehat{Y}_{ct}$ can be constructed as follows:

$$\begin{aligned} (\widehat{\eta}_{ct}^-, \widehat{\eta}_{ct}^+) &= \widehat{\eta}_{ct} \pm Z_\alpha \widehat{S}_{ct} \\ (\widehat{\mu}_{ct}^-, \widehat{\mu}_{ct}^+) &= \text{expit}(\widehat{\eta}_{ct} \pm Z_\alpha \widehat{S}_{ct}) \quad \text{(S8)} \\ (\widehat{Y}_{ct}^-, \widehat{Y}_{ct}^+) &= N_{ct}\text{expit}(\widehat{\eta}_{ct} \pm Z_\alpha \widehat{S}_{ct}) \end{aligned}$$

(S9)

where Z_α is the appropriate quantile of a standard Normal distribution for the desired level of confidence $1-\alpha$.

In-sample prediction intervals can be constructed as:

$$(Y_{ct}^-, Y_{ct}^+) = N_{ct}\text{expit}\left(\widehat{\eta}_{ct} \pm Z_\alpha \sqrt{\frac{1}{N_{ct}\widehat{\mu}_{ct}(1-\widehat{\mu}_{ct})} + \widehat{S}_{ct}^2}\right) . \quad \text{(S10)}$$

Aggregating over fitted values

We need to aggregate

- over time when computing observed and expected values over periods of multiple weeks,
- over countries when computing the expected total numbers of cases arriving at the border from all countries combined.

Given a set of independent Binomial random variables

$$Y_k | N_k, \mu_k \sim \text{Binomial}(N_k, \mu_k) \quad \text{(S11)}$$

where we have estimated $\eta_k = \text{logit}(\mu_k)$ with fitted values $\widehat{\eta}_k = \text{logit}(\widehat{\mu}_k)$ and variances $\text{Var}[\widehat{\eta}_k] = \widehat{S}_k^2$, we can write

$$\sigma_k^2 = N_k \mu_k (1 - \mu_k) \qquad \text{and} \qquad \widehat{\sigma}_k^2 = N_k \widehat{\mu}_k (1 - \widehat{\mu}_k)$$

Aggregation then proceeds as follows:

$$\begin{aligned}
Y &= \sum_k Y_k \\
N &= \sum_k N_k \\
E[Y|\{\widehat{\mu}_k\}] &= \sum_k N_k \widehat{\mu}_k \\
\text{Var}[Y|\{\widehat{\mu}_k\}] &= \sum_k N_k \widehat{\mu}_k (1 - \widehat{\mu}_k) \\
E[\text{Var}[Y|\{\widehat{\mu}_k\}]] &= \sum_k \widehat{\sigma}_k^2 - \sum_k \frac{1}{N_k} \widehat{\sigma}_k^4 \widehat{S}_k^2 \qquad \text{(S12)} \\
\text{Var}[E[Y|\{\widehat{\mu}_k\}]] &= \sum_k \widehat{\sigma}_k^4 \widehat{S}_k^2 \\
\text{Var}[Y] &\simeq \sum_k \widehat{\sigma}_k^4 \widehat{S}_k^2 + \sum_k \widehat{\sigma}_k^2
\end{aligned}$$

So that if we set $\widehat{\mu} = Y/N$ then the variances of needed for confidence and prediction intervals for $\text{logit}(Y/N)$ are, respectively:

$$\text{Var}\left[E\left[\text{logit}\,\frac{Y}{N}\middle|\,\{\widehat{\mu}_k\}\right]\right] = \frac{\sum_k \widehat{\sigma}_k^4 \widehat{S}_k^2}{N^2 \widehat{\mu}^2 (1 - \widehat{\mu})^2} \qquad \text{(S13)}$$

$$\text{Var}\left[\text{logit}\,\frac{Y}{N}\middle|\,\{\widehat{\mu}_k\}\right] = \frac{\sum_k \left(\widehat{\sigma}_k^4 \widehat{S}_k^2 + \widehat{\sigma}_k^2\right)}{N^2 \widehat{\mu}^2 (1 - \widehat{\mu})^2} \;. \qquad \text{(S14)}$$

Forecasts

To forecast the model a further k time steps beyond the last observation t_n, we require first a method for forecasting the number of arrivals N_{ct} and the case rate in country of origin I_{ct}.

- We forecast **arrivals** N_{ct} using known MIQ bookings, or if these are not available, we set $N_{ct} = \bar{N}_c$ for all $t > t_n$ where $\bar{N}_c$ is the mean number of arrivals for country c in a fixed window prior to the last observation at t_n.

- We forecast the **case rate** in country of origin using a weighted linear fit to $\text{logit}(\delta + I_{ct})$ using the last $m = 3$ observations with weights $1/m, 2/m, \ldots, m/m$. δ is again a small offset (10^{-7}) added to all case rate observations to avoid zeros.

We then need to forecast the other components of the model:

- The fixed effect parameters $\widehat{\boldsymbol{\beta}}$ and covariances $\widehat{V}_\beta$ are time invariant;
- The country level random effect errors $\widehat{\sigma}_u$ are time invariant;
- The AR(1) correlation and error $\widehat{\rho}$, $\widehat{\sigma}_e$ are time invariant;
- The country level random effects $\widehat{u}_c$ are time invariant;
- The country-period level random effects, predicting k steps forward:

$$\widehat{v}_{ct_{n+k}} = \widehat{\rho}^k v_{ct_n} + \zeta_{ct_{n+k}}$$

  where

$$\zeta_{ct_{n+k}} \overset{\text{iid}}{\sim} N\left(0, \widehat{\sigma}_e^2 \frac{1 - \widehat{\rho}^{2k}}{1 - \widehat{\rho}^2}\right)$$

  Forecasts use the expected value $\widehat{v}_{ct_{n+k}} = \widehat{\rho}^k v_{ct_n}$.

Conveniently, forecasts of the infection rates of arrivals on the logit scale ($\widehat{\eta}_{ct}$) and their associated standard errors ($\widehat{S}_{ct}$) can be computed when the model is being fitted at the estimation stage. In this procedure we set both the observed numbers of arrivals (N_{ct}) and numbers of observed cases (Y_{ct}) to zero for the time periods to be forecasted, and include them in the data set during the fitting process. The likelihood contributions from these observations are all independent of the parameters, and do not affect the estimation process. These time periods are thus treated in the same way as time periods that were truly observed within the data set, but at which no arrivals occurred.

When estimating confidence and prediction intervals for the future observations, we replace the zero values of the arrivals N_{ct} with the forecast arrival numbers, and then use the methods of the Estimation section above.

Note that these confidence and prediction intervals both neglect any uncertainty introduced in the forecasting of the numbers of arrivals and of the case rate in country of origin. The number of arrivals may be estimated from Managed Isolation and Quarantine (MIQ) bookings, and for instances where passengers are not required to use MIQ estimates can be made from flight schedules.

One step ahead forecasts

One step ahead forecasts are useful measures of goodness of fit, and were used in the model selection procedure described in the main text and below.

On the logit scale the one step ahead forecast is

$$\widehat{\eta}_{ct|t-1} = \mathbf{I}_{ct}^T \widehat{\boldsymbol{\beta}} + \widehat{u}_c + \widehat{\rho}\widehat{v}_{c,t-1} \tag{S15}$$

with variance

$$\mathrm{Var}[\widehat{\eta}_{ct|t-1}] = \mathrm{Var}[\mathbf{I}_{ct}^T \widehat{\boldsymbol{\beta}} + \widehat{u}_c + \widehat{\rho}\widehat{v}_{c,t-1}] \tag{S16}$$

which we estimate by its approximate upper bound

$$\mathrm{Var}[\widehat{\eta}_{ct|t-1}] \leq \widehat{S}_{c,t-1}^2 + \max\left(0, \mathbf{x}_{ct}^T \widehat{V}_\beta \mathbf{I}_{ct} - \mathbf{x}_{c,t-1}^T \widehat{V}_\beta \mathbf{I}_{c,t-1}\right) \ . \tag{S17}$$

Low information countries

We cannot make good estimates using the above model for countries where there is low information, either due to low numbers of arrivals, low numbers of cases, or both.

The model described above is fitted only for countries with 50 or more arrivals and 5 or more cases in the most recent 30 weeks.

The fitted model has estimates of the parameters ($\boldsymbol{\beta}$, σ_u, σ_e, ρ) and the random effects u_c, v_{ct} for the modelled countries only. The fitted model also estimates the variance-covariance of the fixed effect parameters $\widehat{V}_\beta$.

Low case numbers

For the remaining countries where there has been at least one arrival ($\sum_t N_{ct} > 0$) and at least one case ($\sum_t Y_{ct} > 0$), we remove the autoregressive AR(1) component from (S2), and fit the simpler model to the data (N_{ct}, y_{ct}):

$$Y_{ct} | N_{ct}, \mu_{ct} \sim \mathrm{Binomial}(N_{ct}, \mu_{ct}) \tag{S18}$$

$$\eta_{ct} = \mathrm{logit}\mu_{ct} = \mathbf{I}_{ct}^T \widehat{\boldsymbol{\beta}} + u_c \tag{S19}$$

where u_c is a (fixed effect) parameter to be estimated, and the term $\mathbf{I}_{ct}^T\widehat{\beta}$ is treated as a known offset (i.e. $\boldsymbol{\beta}$ is not re-estimated).

We set u_c to the fitted value $\widehat{u}_c$ from the fitted model, and set all $v_{ct} = 0$. Thus

$$\begin{aligned} \widehat{\eta}_{ct} &= \mathbf{I}_{ct}^T\widehat{\boldsymbol{\beta}} + \widehat{u}_c & \text{(S20)} \\ \text{Var}[\widehat{\eta}_{ct}] = \widehat{S}_{ct}^2 &\simeq \mathbf{x}_{ct}^T\widehat{V}_\beta\mathbf{I}_{ct} + \text{Var}[\widehat{u}_c] & \text{(S21)} \end{aligned}$$

where $\widehat{\boldsymbol{\beta}}$ and $\widehat{V}_\beta$ come from the original fit and $\text{Var}[\widehat{u}_c]$ comes from the fit to the low case data.

Forecasting k time steps beyond the final observation at t_n uses the main model estimates of the AR(1) correlation $\widehat{\rho}$ and variance $\widehat{\sigma}_e^2$:

$$\begin{aligned} \widehat{\eta}_{ct_{n+k}} &= \mathbf{I}_{ct_{n+k}}^T\widehat{\boldsymbol{\beta}} + \widehat{u}_c & \text{(S22)} \\ \text{Var}[\widehat{\eta}_{ct_{n+k}}] = \widehat{S}_{ct_{n+k}}^2 &\simeq \mathbf{x}_{ct_{n+k}}^T\widehat{V}_\beta\mathbf{I}_{ct_{n+k}} + \text{Var}[\widehat{u}_c] + \widehat{\sigma}_e^2\frac{1-\widehat{\rho}^{2k}}{1-\widehat{\rho}^2} & \text{(S23)} \end{aligned}$$

These formulae apply to all forecasts (including the one step ahead forecast $\widehat{\eta}_{ct|t-1}$).

Zero cases

For all other countries (where there have been zero cases) we set $u_c = 0$ and $v_{ct} = 0$. With no further model fitting we simply set

$$\begin{aligned} \widehat{\eta}_{ct} &= \mathbf{I}_{ct}^T\widehat{\beta} \\ \text{Var}[\widehat{\eta}_{ct}] = \widehat{S}_{ct}^2 &\simeq \mathbf{x}_{ct_n}^T\widehat{V}_\beta\mathbf{I}_{ct_n} \end{aligned}$$

where $\widehat{\boldsymbol{\beta}}$ and $\widehat{V}_\beta$ come from the original main model fit.

Forecasting k time steps beyond the final observation at t_n uses the estimates of the AR(1) correlation $\widehat{\rho}$ and variance $\widehat{\sigma}_e^2$:

$$\begin{aligned} \widehat{\eta}_{ct_{n+k}} &= \mathbf{I}_{ct_{n+k}}^T\widehat{\boldsymbol{\beta}} & \text{(S24)} \\ \text{Var}[\widehat{\eta}_{ct_{n+k}}] = \widehat{S}_{ct_{n+k}}^2 &\simeq \mathbf{x}_{ct_{n+k}}^T\widehat{V}_\beta\mathbf{I}_{ct_{n+k}} + \widehat{\sigma}_e^2\frac{1-\widehat{\rho}^{2k}}{1-\widehat{\rho}^2} & \text{(S25)} \end{aligned}$$

These formulae apply to all forecasts (including the one step ahead forecast $\widehat{\eta}_{ct|t-1}$).

Model selection

In this section we describe the model selection procedure. The available predictor variables are, for time period t and country c:

- Death rate, D_{ct}
- Case rate in country of origin, I_{ct}
- Prevalence rate, P_{ct}
- Test rate, TR_{ct}
- Test positivity rate, TP_{ct}
- Partial vaccination rate, PV_{ct}
- Fully vaccinated rate, FV_{ct}
- Effective Reproduction number, R_{ct}

All predictors are available at each time period. Where daily estimates are available we aggregate to weekly values by taking the weekly median. We use $\mathbf{x}_{ct}$ to denote the vector of all predictors for country c at time t.

We show the model selection process here for a data set restricted to arrivals in the 30 week period starting 26 April 2021 and ending on 21 November 2021. In this period there were 388 cases among 53275 arrivals from 185 countries. We further restrict modelling to countries where there were at 5 or more cases arriving during the period. This reduces the data to 298 cases among 32825 arrivals from 17 countries. These are 62% of the total number of arrivals, and 77% of the cases. (The list of modelled countries is Afghanistan, Australia, Fiji, India, Indonesia, Iraq, Japan, Malaysia, Philippines, Qatar, Russia, Singapore, South Africa, Sri Lanka, United Arab Emirates, United Kingdom, United States.)

When comparing time series models where prediction is the goal, prediction errors are typically used rather than standard (relative) goodness of fit measures such as AIC. The use of AIC risks overfitting the model to the observed sample data, rather than minimising the error of predictions. Here we use the mean absolute deviation (MAD) of one step ahead prediction errors to measure goodness of fit, and as the criterion for model selection when determining which predictors to include in the final model.

When fitting the model we estimate the linear predictor

$$\widehat{\eta}_{ct} = \mathbf{x}_{ct}^T\widehat{\boldsymbol{\beta}} + \widehat{u}_c + \widehat{v}_{ct}$$

at each time point for each country, along with its standard error $\widehat{S}_{ct}$. The one step ahead forecast is

$$\widehat{\eta}_{ct|t-1} = \mathbf{x}_{ct}^T\widehat{\boldsymbol{\beta}} + \widehat{u}_c + \widehat{\rho}\widehat{v}_{c,t-1}$$

with approximate estimation standard error $\widehat{S}_{ct|t-1} = \sqrt{\widehat{S}_{ct}^2 + \widehat{\sigma}_e^2}$.

On the observation scale the fitted value and one step ahead fitted value are

$$\begin{aligned}\widehat{Y}_{ct} &= N_{ct}\text{expit}(\widehat{\eta}_{ct})\\ \widehat{Y}_{ct|t-1} &= N_{ct}\text{expit}(\widehat{\eta}_{ct|t-1})\end{aligned}$$

The mean absolute deviation is then defined as

$$\text{MAD} = \frac{1}{CT}\sum_{ct}|Y_{ct} - \widehat{Y}_{ct|t-1}|$$

where p is the number of parameters in the model, C is the number of countries and T is the number of weeks. We omit from the sum (and adjust the $1/(CT)$ denominator) any observations where $N_{ct} = 0$, and for which therefore $\widehat{Y}_{ct|t-1} = 0$.

In order to compare models we need a subset of the data for which all the potential covariates are present. There is a substantial amount of missing data in vaccine coverage and population testing, with some countries not reporting these data consistently, and in some cases not all.

We tested two data sets in selecting the model: each one 30 weeks long. The first contains weeks 38 to 67 (28 Sep 2020-25 Apr 2021) and the second weeks 68-97 (26 Apr 2021-21 Nov 2021). In each data set we restricted to countries with 50 or more arrivals (22 countries in the first data set, and 17 in the second). In any instances country/week combinations where any covariates were missing we set the number of arrivals and cases for that country in that week to zero. This means that those rows did not influence the estimation process.

We carried out forward and backwards model selection, analogous to model selection by an information criterion such as AIC. In the forward selection procedure we started with a model which included no covariates and then added covariates to the model one by one, as long as the MAD value decreased. At each step we selected the covariate that resulted in the greatest reduction in MAD. The backwards model selection proceeded similarly, but starting with the model with all covariates, successively deleting the one which led to the greatest reduction in MAD, and terminating when no further removals reduced the MAD.

Table S2: Model selection by MAD. A blank indicates that the relevant covariate was not included in the final model. Data Set 1 covers 28 Sep 2020-25 Apr 2021 and Data Set 2 covers 26 Apr 2021-21 Nov 2021. ($\delta = 10^{-7}$ is a small offset to prevent taking the log of zero.)

	Data Set 1; forwards		Data Set 1: backwards		Data Set 2: forwards		Data Set 2: backwards	
Parameter	Estimate	Std. Err.	Estimate	Std. Err.	Estimate	Std. Err.	Estimate	Std. Err.
(Intercept)	-7.80	1.6731	-7.80	1.6731	-2.287	0.998	-2.522	1.083
logit($\delta + I_{c,t}$)	1.11	0.6721	1.11	0.6721			-1.461	1.556
logit($\delta + I_{c,t-1}$)	-0.54	0.8885	-0.54	0.8885			2.407	1.393
logit($\delta + I_{c,t-2}$)							-1.366	1.239
logit($\delta + I_{c,t-3}$)	0.81	0.4672	0.81	0.4672	0.434	0.132	0.848	0.783
logit($\delta + D_{c,t}$)	-1.11	0.2540	-1.11	0.2540				
logit($\delta + PV_{ct}$)							0.255	0.370
logit($\delta + FV_{ct}$)	-0.0493	0.0421	-0.0493	0.0421	-0.341	0.113	-0.568	0.378
log(R_{eff})							1.049	2.706
σ_u	9.33e-05	0.2975	9.33e-05	0.2975	0.404	0.312	0.308	0.536
σ_e	1.14	0.1771	1.14	0.1771	1.266	0.194	1.283	0.242
ρ	0.496	0.1270	0.496	0.1270	0.430	0.163	0.438	0.172
AIC	355		355		666		672	
MAD	0.971		0.971		0.620		0.614	

Model selection results

The results of the forward and backward model selection algorithms applied to the two data sets are shown in Table S2. The forward and backward methods select the same model for Data Set 1, whereas there are some differences for Data Set 2, with the backwards method preferring a more complex model.

For comparison in Table S3, the parameter estimates, AIC and MAD values are shown for a model including only case rate in country of origin at lags 0, 1 and 2, and no other covariates. The MAD values differ very little from any of those in Table S2, and are therefore just as good as those more complex models from the point of view of one step prediction.

The case rate only models also have the advantage that they rely only on covariates that are

Table S3: Parameter estimates for a model with three lags of case rate as the only predictors to the two data sets.

	Data Set 1		Data Set 2	
Parameter	Estimate	Std. Err.	Estimate	Std. Err.
α	-1.779	1.448	-2.444	1.109
β_0	1.081	0.724	-0.756	0.687
β_1	-0.936	1.055	2.034	1.222
β_2	0.267	0.708	-0.938	0.715
σ_u	0.358	1.931	0.771	0.247
σ_e	1.426	0.499	1.173	0.186
ρ	0.785	-0.256	0.485	0.161
AIC	367.311		672.092	
MAD	1.073		0.637	

Table S4: Goodness of fit for models from Table S3 with and without random effects

	Data Set 1		Data Set 2	
Model	AIC	MAD	AIC	MAD
No Random Effects	656	1.38	928	0.844
+ Country Level RE	440	1.04	727	0.663
+ AR(1) Correlation	367	1.07	672	0.637

readily available, and are the most complete both across countries and across time. For this reason we have opted to use the three lag case rate modelling for our border risk assessment.

Table S4 shows the effect on the values of AIC and MAD of successively adding country level and the autoregressive random effects to a model with lagged case rate in country of origin as the only predictors. The inclusion of random effects at country level is strongly supported by both AIC and MAD. The justification for serial correlation is weaker using the MAD criterion: being weakly supported in Data Set 2, but not in Data Set 1. We have nevertheless retained the serial correlation in the main model in order to allow for short term excursions in infection risk.

Supplementary figures

Figure S2 shows cases per thousand in the country of origin and rates of infection among arrivals to New Zealand in the 19 modelled countries (those with at least 50 arrivals and at least 5 cases).

The numbers of cases and the modelled estimates are shown in Figure S3 and the corresponding border infection rates are shown in Figure S4. For each country a 5 week forecast period is shown with the actual counts/rates shown as white squares.

Figure S5 displays the estimate value of the time independent ($\widehat{u}_c$) and autoregressive ($\widehat{v}_{ct}$) random effects for each of the 10 modelled countries. In the five week forecast period, and in any period for which there are no arrivals, the autoregressive component declines to zero, leaving only the estimated country level effect u_c. In countries such as the United Kingdom and United States, the combined random effects $\widehat{u}_c + \widehat{v}_{ct}$ are consistently negative, indicating that travellers have lower rates of infection than the general population level risk in those countries. For India, Indonesia and Iraq the random effects are consistently positive, showing an apparent increased risk than the source country population rate of infection would indicate. Such instances are likely to be caused by underreporting of cases.

Figures S7 and S8 show the arrivals N_{ct} and incidence rates I_{ct} and their forecast values $\widehat{N}_{ct}$ and $\widehat{I}_{ct}$.

References

[1] RNZ. NZ to close doors on foreign travellers from China; 2020. Available from: https://www.rnz.co.nz/news/national/408675/nz-to-close-doors-on-foreign-travellers-from-china.

[2] Cooke H. Coronavirus: Government shuts borders to all but citizens and residents; 2020. Available from: https://www.stuff.co.nz/national/health/coronavirus/120423903/coronavirus-prime-minister-to-make-announcement-on-border-controls.

[3] Ministry of Health. History and origins of MIQ; 2022. Available from: https://www.miq.govt.nz/about/history-and-origins-of-miq.

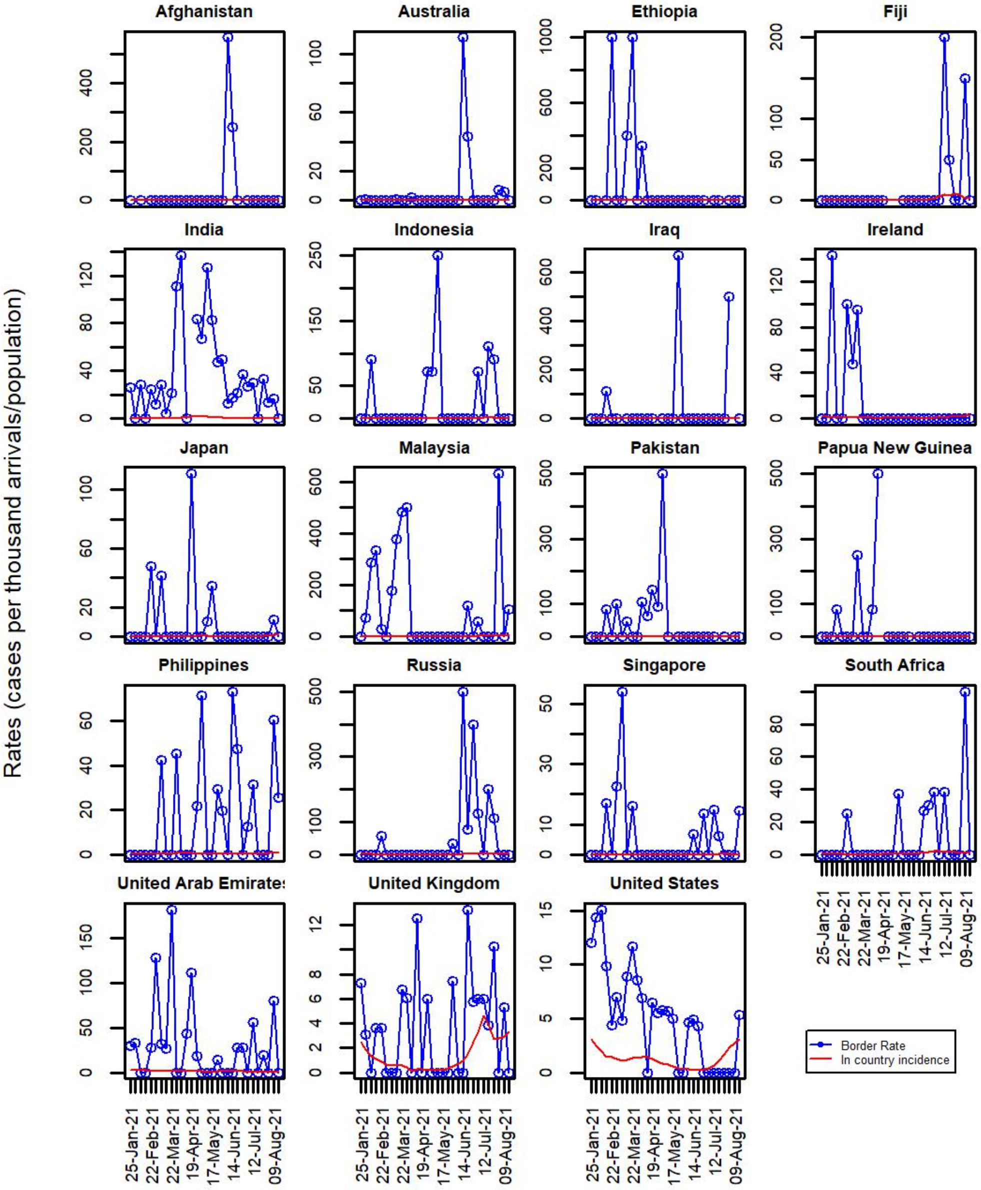

Figure S2: Data on weekly reported cases per thousand in the country of origin (solid red curves) and observed rates of infection among arrivals to New Zealand (blue curves with open circles) in the 19 fully modelled countries.

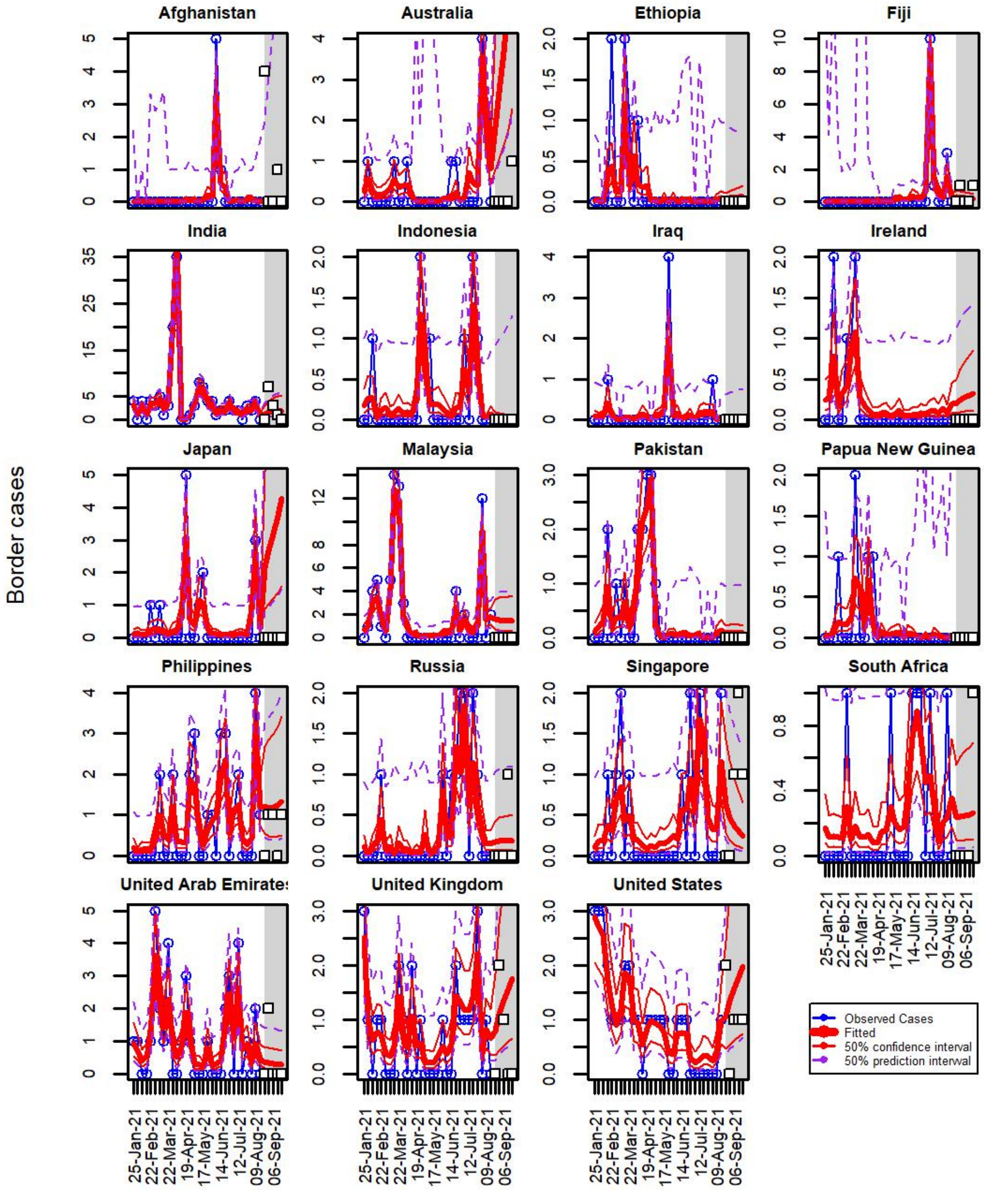

Figure S3: Number of arriving cases from each of the 19 fully modelled countries (blue). The thick red lines show the fitted model, the thin red lines show the 50% confidence interval around this fit, and the purple dashed lines show the 50% prediction interval. Model fitted to data from 25 January to 22 August 2021. Shaded grey region shows a 12 week forecast period alongside actual data for the first 5 weeks of the forecast period (white squares).

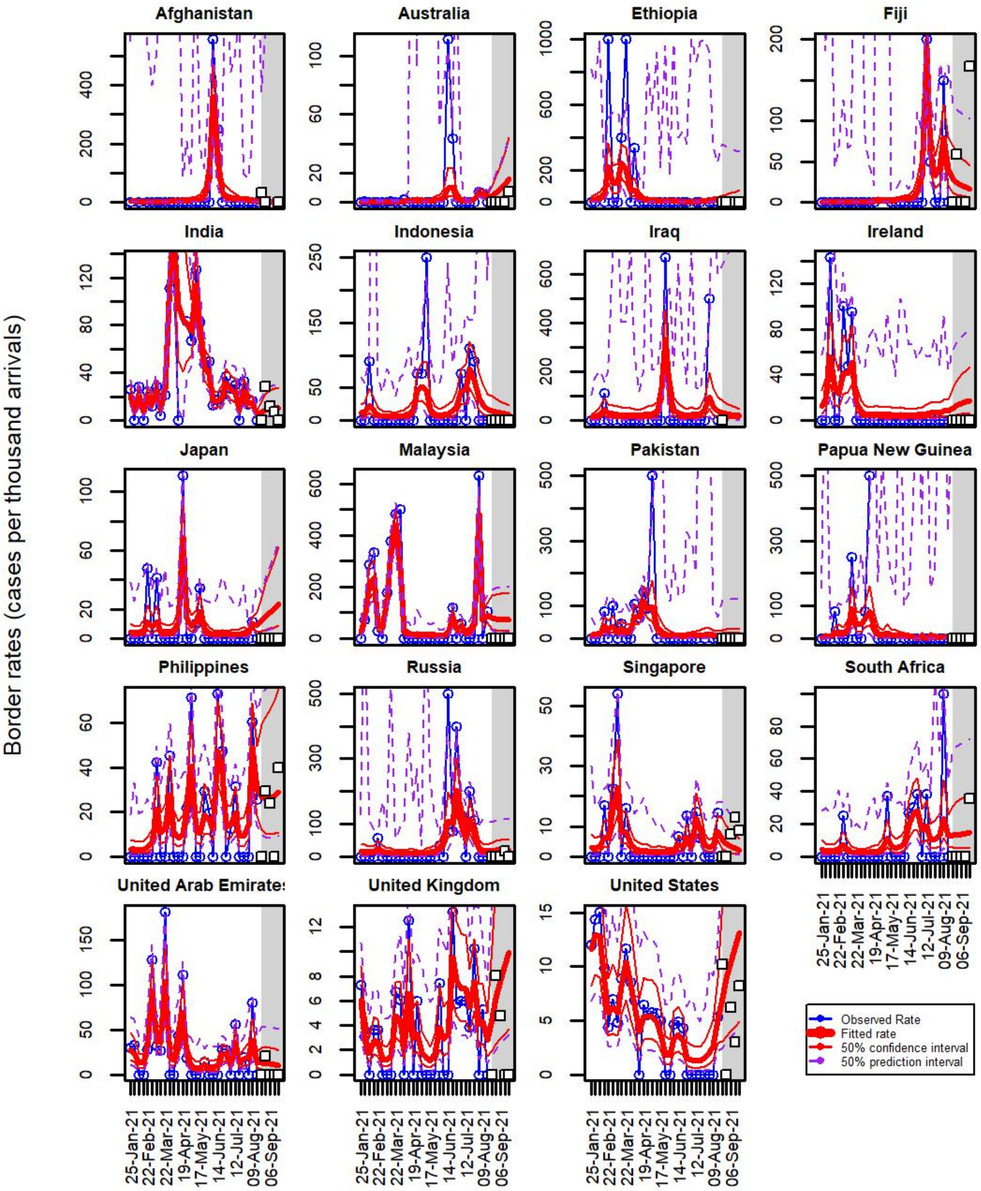

Figure S4: Infection rates at the New Zealand border (new cases per week per thousand arrivals) in the fully modelled countries. The thick red lines show the fitted model, the thin red lines are a 50% confidence interval around this fit. The purple dashed lines are a 50% prediction interval. The grey shaded area at right shows the predictions of the model, and the white squares are the actual observations in the 5 weeks following the period of the training data.

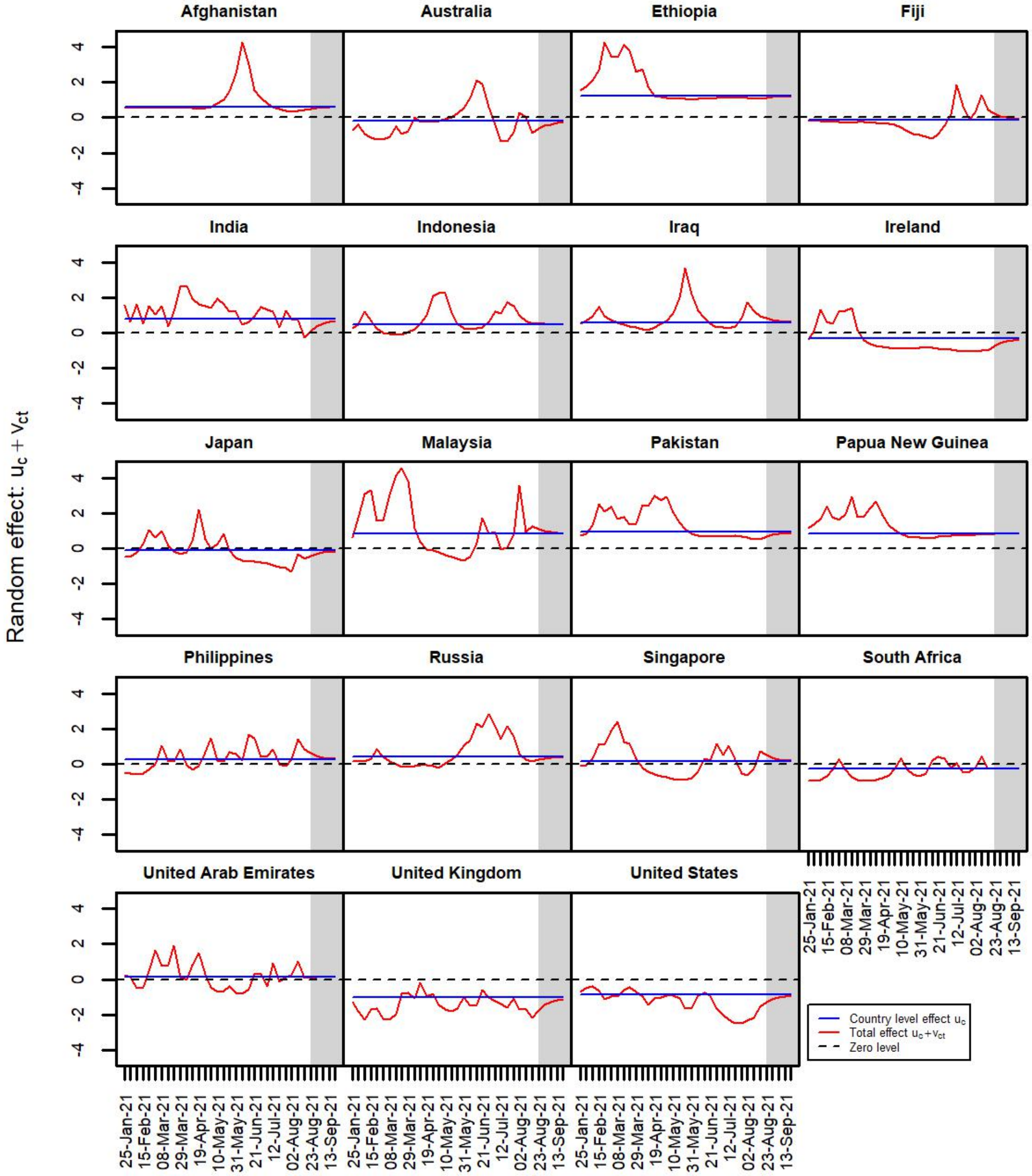

Figure S5: Time dependence of the random effects $u_c + v_{ct}$ in the fully modelled countries. The blue line shows the time independent country-level random effect u_c, and the red line is the full time dependent random effect $u_c + v_{ct}$. The dashed line at zero is the mean of the country level random effects. Travellers from countries with consistently positive random effects have **higher** rates of infection than the incidence in the general population would predict. Negative random effects imply lower rates of infection than the general population. In the grey shaded area where predictions are made the time dependent part of the random effect v_{ct} decays exponentially to zero.

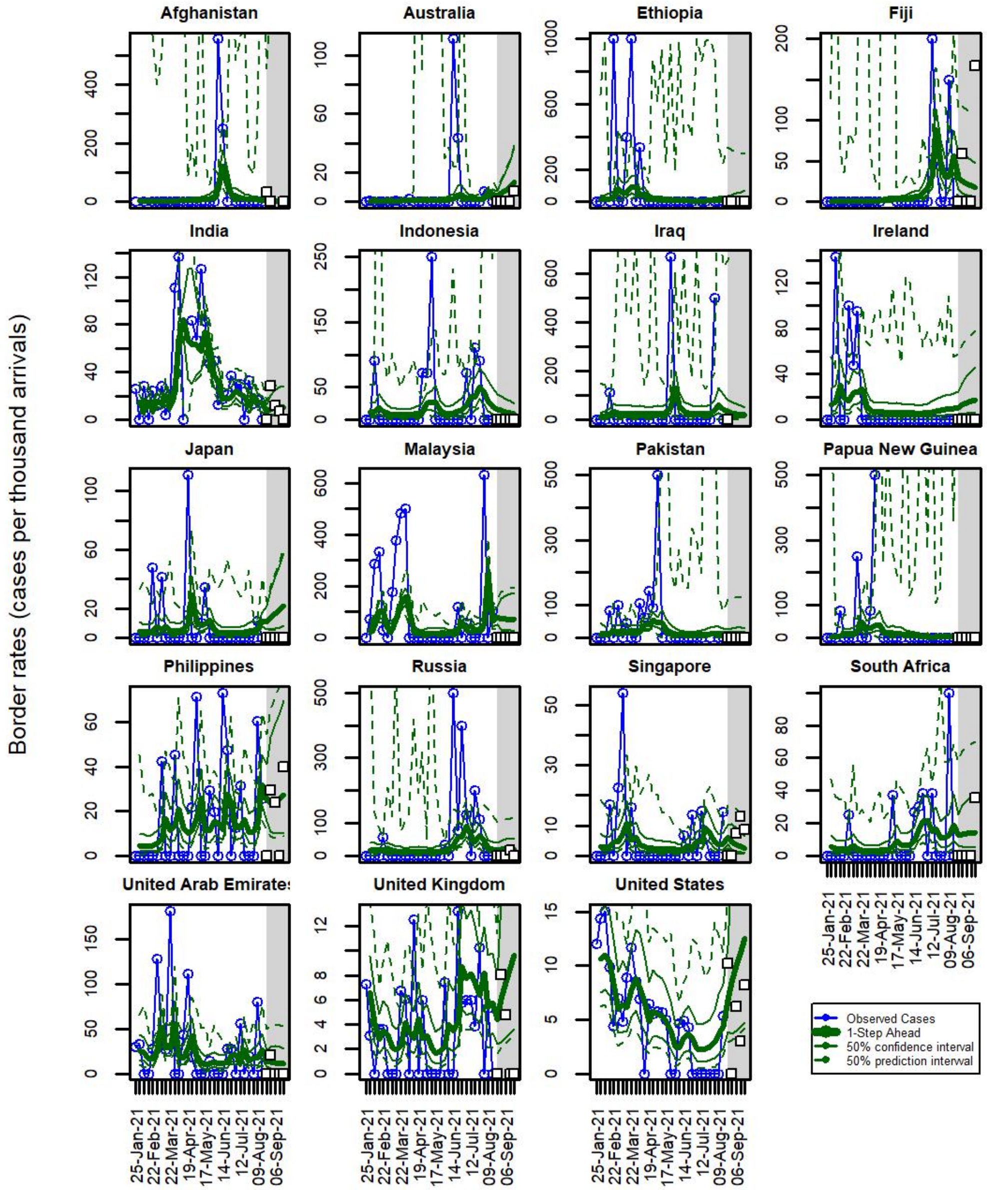

Figure S6: One step ahead predictions for infection rates among arriving cases from each of the 19 fully modelled countries. Observed rates are shown in blue, the thick green lines show the one step ahead predictions with 50% confidence intervals (thin green lines) and 50% prediction intevals (dashed green lines). Model fitted to data from 25 January to 22 August 2021. Shaded grey region shows a 5 week forecast period alongside actual data for the first 5 weeks of the forecast period (white squares).

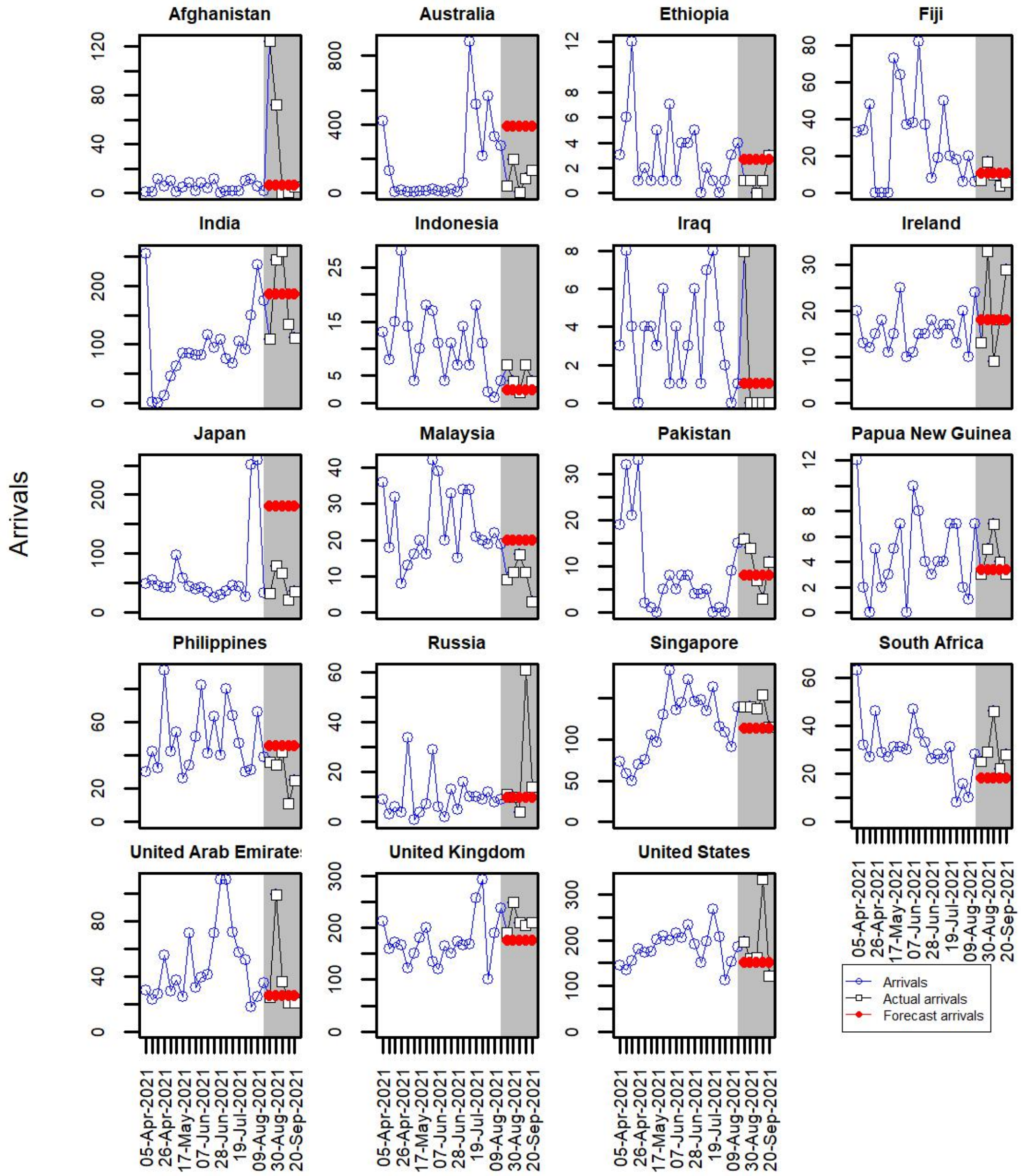

Figure S7: Actual and forecast arrivals for the 19 fully modelled countries. 20 weeks of arrivals used in the training period are shown in blue, and then the 5 week forecast period shaded in grey. The actual arrivals are shown by white squares, and the forecast arrivals (always a mean of the last three weeks) are shown with red filled circles.

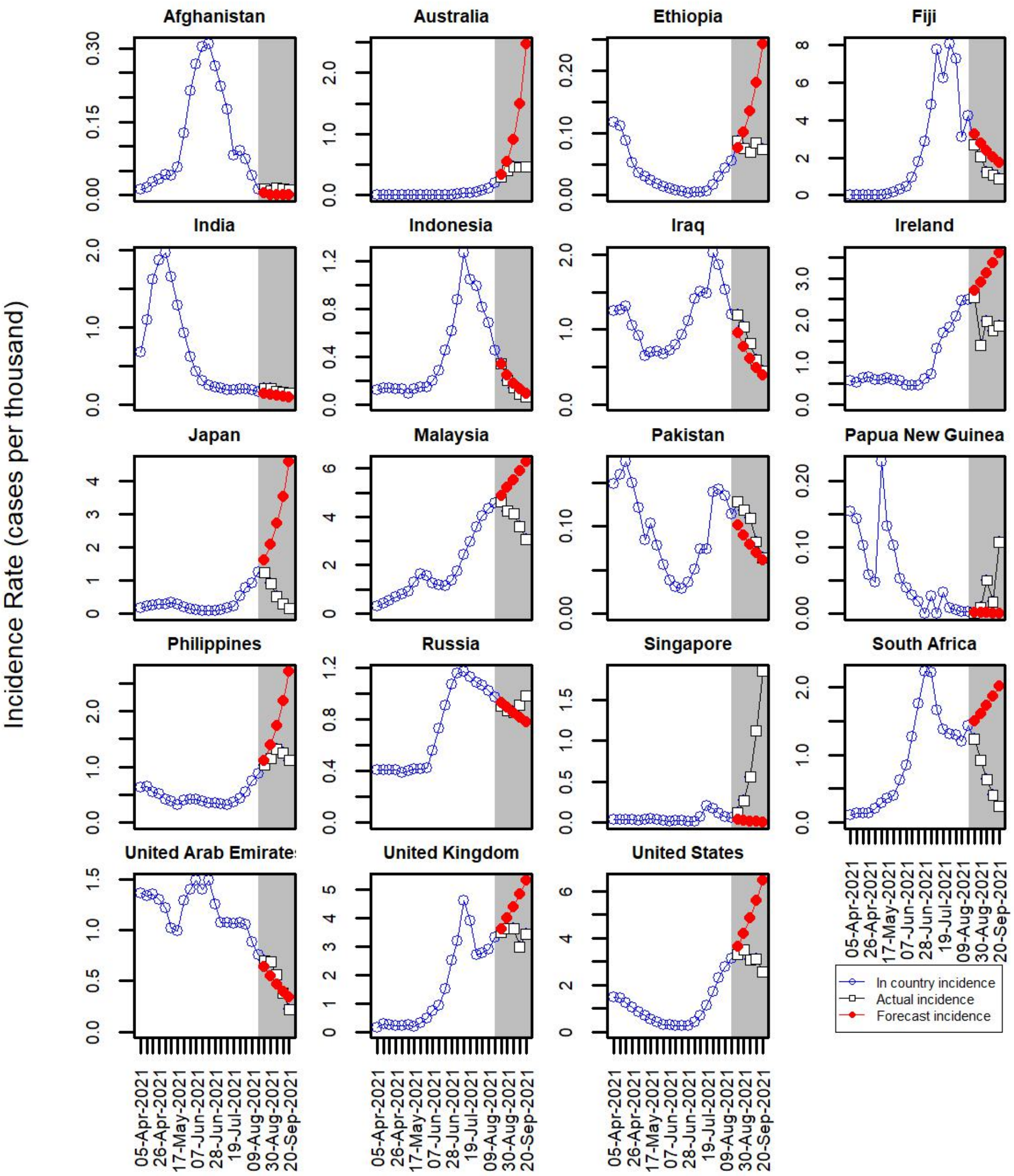

Figure S8: Actual and forecast in-country incidence rates for the 19 fully modelled countries. 20 weeks of in-country incidence rates used in the training period are shown in blue, and then the 5 week forecast period shaded in grey. The actual incidence rates are shown by white squares, and the forecast incidence rates (a log linear extrapolation of the last three weeks) are shown with red filled circles.

[4] One News. Isolation hotel vouchers now required to enter NZ, with pre-Christmas period completely booked; 2020. Available from: https://www.tvnz.co.nz/one-news/new-zealand/isolation-hotel-vouchers-now-required-enter-nz-pre-christmas-period-completely-booked.

[5] RNZ. Covid-19: UK and US travellers to need pre-departure tests before departing to NZ; 2021. Available from: https://www.rnz.co.nz/news/national/433968/covid-19-uk-and-us-travellers-to-need-pre-departure-tests-before-departing-to-nz.

[6] NZ Herald. Covid 19 coronavirus: Government announces new Covid testing rules; 2021. Available from: https://www.nzherald.co.nz/nz/covid-19-coronavirus-government-announces-new-covid-testing-rules/C4QQPKH2I6J7HZC3ODHBJBNTOY.

[7] Early M. Covid-19: More people now eligible for emergency slots in managed isolation; 2021. Available from: https://www.stuff.co.nz/national/health/coronavirus/300267231/covid19-more-people-now-eligible-for-emergency-slots-in-managed-isolation.

[8] Newshub. Coronavirus: Jacinda Ardern announces suspension of arrivals from India for two weeks; 2021. Available from: https://www.newshub.co.nz/home/politics/2021/04/coronavirus-stopping-flights-from-india-not-ruled-out-as-cases-in-miq-from-asian-nation-jump.html.

[9] NZ Herald. New Zealand and Australia travel bubble to begin on April 19 - Prime Minister Jacinda Ardern; Air NZ 'run off our feet'; 2021. Available from: https://www.nzherald.co.nz/nz/new-zealand-and-australia-travel-bubble-to-begin-on-april-19-prime-minister-jacinda-ardern-air-nz-run-off-our-feet/CE5FN7UHT23NEY2VVKQZWANFQI/.

[10] Ardern J, Hipkins C. Quarantine Free Travel with Australia suspended; 2021. Available from: https://www.beehive.govt.nz/release/quarantine-free-travel-australia-suspended.

[11] Hipkins C. Making the border stronger; 2021. Available from: https://www.beehive.govt.nz/release/making-border-stronger.

[12] Immigration New Zealand. Restriction on travel from very high risk countries (28 Aug 2021); 2021. Available from: https://web.archive.org/web/20210828043928/https://www.immigration.govt.nz/about-us/covid-19/border-closures-and-exceptions/restrictions-on-travel-from-very-high-risk-countries.

[13] Hipkins C. Fiji and Indonesia designated very high risk countries due to COVID-19 risk; 2021. Available from: https://www.beehive.govt.nz/release/fiji-and-indonesia-designated-very-high-risk-countries-due-covid-19-risk.

[14] Manch T. Government will require non-NZ citizens to have Covid vaccine before entering New Zealand; 2021. Available from: https://www.stuff.co.nz/national/health/coronavirus/126566572/government-will-require-nonnz-citizens-to-have-covid-vaccine-before-entering-new-zealand.

[15] RNZ. Covid-19: Major MIQ changes from early next year; 2021. Available from: https://www.rnz.co.nz/news/national/456430/covid-19-major-miq-changes-from-early-next-year.

[16] Brooks ME, Kristensen K, van Benthem KJ, Magnusson A, Berg CW, Nielsen A, et al. glmmTMB Balances Speed and Flexibility Among Packages for Zero-inflated Generalized Linear Mixed Modeling. The R Journal. 2017;9(2):378–400. Available from: https://journal.r-project.org/archive/2017/RJ-2017-066/index.html.

[17] Kristensen K, Nielsen A, Berg CW, Skaug H, Bell BM. TMB: Automatic Differentiation and Laplace Approximation. Journal of Statistical Software. 2016;70(5):121. Available from: https://www.jstatsoft.org/index.php/jss/article/view/v070i05.
